# Fluorohydration of alkynes via I(I)/I(III) catalysis

**DOI:** 10.3762/bjoc.16.135

**Published:** 2020-07-10

**Authors:** Jessica Neufeld, Constantin G Daniliuc, Ryan Gilmour

**Affiliations:** 1 Organisch-Chemisches Institut, Westfälische Wilhelms-Universität Münster, Corrensstraße 40, 48149 Münster, Germany

**Keywords:** α-fluoroketone, alkyne, fluorination, hypervalent iodine, organocatalysis

## Abstract

Substrate specificity is ubiquitous in biological catalysis, but less pervasive in the realm of small-molecule catalysis. Herein, we disclose an intriguing example of substrate specificity that was observed whilst exploring catalysis-based routes to generate α-fluoroketones from terminal and internal alkynes under the auspices of I(I)/I(III) catalysis. Utilising *p*-TolI as an inexpensive organocatalyst with Selectfluor^®^ and amine/HF mixtures, the formation of protected α-fluoroketones from simple alkynes was realised. Whilst the transient *p*-TolIF_2_ species generated in situ productively engaged with pentynyl benzoate scaffolds to generate the desired α-fluoroketone motif, augmentation or contraction of the linker suppressed catalysis. The prerequisite for this substructure was established by molecular editing and was complemented with a physical organic investigation of possible determinants.

## Introduction

**Figure 1:**
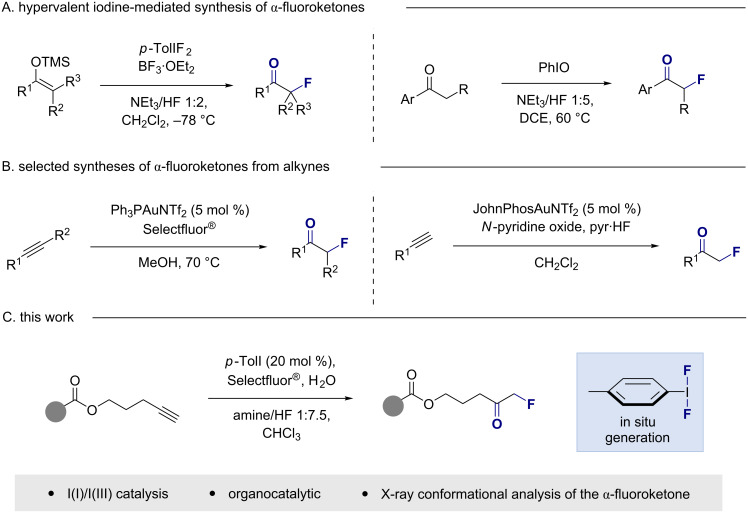
(A) Synthetic routes to α-fluoroketones from silyl enol ethers or acetophenone derivatives. (B) Selected Au-catalysed syntheses of α-fluoroketones from alkynes. (C) This work: synthesis of α*-*fluoroketones from pentynyl benzoates via I(I)/I(III) catalysis.

The venerable role of fluorine as a powerful physicochemical modulator in small-molecule drug discovery is a compelling argument for the importance of sustained innovation to facilitate this form of structural editing [1–4]. Routinely employed as a bioisostere of hydrogen or hydroxy [5–6], substitution may enable inversion of the local electronic environment, or the precise deletion of hydrogen-bond donors, respectively, with minimal steric variation [7]. The latter aspect is a crucial determinant in enabling fluorinated materials to adopt intriguing conformations which are a manifest representation of stabilising non-covalent interactions [8–10]. These collective attributes render fluorination a valuable strategy in drug discovery [11] and agrochemical development [12]. In the conception of enabling fluorination technologies, the α-fluorocarbonyl motif has emerged as a prominent target scaffold [13]. Remarkable advances in the direct fluorination (F_2_/N_2_) of carbonyl compounds by Sandford and co-workers [14–15] are noteworthy in this regard, and mitigate the expense and poor atom economy associated with stoichiometric approaches. Moreover, this substructure provides a versatile entry point for subsequent C(sp^3^)–C(sp^3^) bond-forming reactions [16–17], and closely resembles one of the very few biological blueprints for the preparation of fluorine-containing materials [18–21]. However, in translating the fluoroacetate-like building blocks to a laboratory paradigm, the intrinsic toxicity of these species in inhibiting aconitase [22] must be reconciled with synthetic utility. To that end, catalysis-based strategies to unmask the venerable α-fluorocarbonyl motif [23] from a terminal alkyne were considered (Figure 1). This general strategy was appealing given the commercial availability of the substrates and the plenum of methods available to activate π-bonds [24–25].

Confidence in this approach stemmed from our recent *vicinal* and *geminal* difluorination of alkenes [26–30], and contemporaneous studies from the Jacobsen laboratory [31–34], under the auspices of I(I)/I(III) catalysis [35–37]. Moreover, elegant studies by Hara and co-workers have demonstrated that α-fluoroketones could be prepared by exposing silyl enol ethers to stoichiometric *p*-ToIIF_2_, in the presence of BF_3_·OEt_2_ and NEt_3_/HF 1:2 [38]. A report by Kitamura and co-workers in which the direct fluorination of acetophenone derivatives was achieved using iodosylarenes and commercially available NEt_3_/HF 1:5 to generate the ArIF_2_ species in situ is also highly pertinent [39–40] (Figure 1A).

Elegant reports describing the conversion of internal alkynes to α-fluoroketones via π-acid catalysis were also disclosed (Figure 1B). Seminal reports by Nevado [41–42] and Gouverneur [43] enabled the synthesis of α-fluoroketones and -enones from terminal and internal alkynes, thereby mitigating difluoroketone formation: This is commonly observed in the fluorination of alkynes using stoichiometric electrophilic fluorinating reagents [44–45]. Developments by Hammond and Xu validated *N*-pyridine oxides as terminal oxidants to substitute Selectfluor^®^ for the cationic Au(I)/Au(III) cycle, thereby enabling high functional group tolerance [46–49]. Inspired by these and other selected advances [50–51], in metal-based fluorination, a complementary organocatalytic variant [52], based on an I(I)/I(III) catalysis platform was explored (Figure 1C). It was envisaged that the in situ generation of *p*-TolIF_2_ via oxidation of *p*-TolI with Selectfluor^®^ in the presence of an amine/HF complex might enable the title transformation [53].

## Results and Discussion

To explore the feasibility of generating α-fluoroketones from terminal alkynes via I(I)/I(III) catalysis, 4-pentynyl benzoate (**1**) was selected as the substrate for optimisation studies (Table 1). The inexpensive, commercially available *p*-TolI was used as an organocatalyst, Selectfluor^®^ was employed as the terminal oxidant, and an amine/HF complex enlisted as the fluoride source (please see Supporting Information File 1 for full details). The reaction was performed in CHCl_3_ at ambient temperature and the crude reaction mixtures were analysed by ^19^F NMR spectroscopy using ethyl fluoroacetate as the internal standard. Initially, the effect of Brønsted acidity was explored by varying the amine/HF ratio with mixtures of commercially available NEt_3_/HF 1:3 and Olah’s reagent (pyr/HF 1:9.23). Lower amine/HF ratios (1:4.5) resulted in poor conversion (9% yield, Table 1, entry 1), whereas increasing the Brønsted acidity to 1:7.5 furnished the desired α-fluoroketone **2** in 64% yield (Table 1, entry 2). However, employing Olah’s reagent (1:9.23) had a negative impact on the efficiency (45% yield, Table 1, entry 3) thereby allowing a plateau to be established (vide infra).

**Table 1:** Reaction optimisation for the synthesis of α-fluoroketone **2** from alkyne **1**.^a^

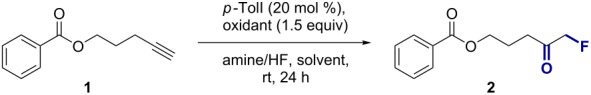				

entry	oxidant	amine/HF^b^	solvent	yield^c^

1	Selectfluor^®^	1:4.5	CHCl_3_	9%
**2**	**Selectfluor**^®^	**1:7.5**	**CHCl_3_**	**64%**
3	Selectfluor^®^	1:9.23	CHCl_3_	45%
4	Selectfluor^®^	1:7.5	CH_2_Cl_2_	56%
5	Selectfluor^®^	1:7.5	HFIP	52%
6	Selectfluor^®^	1:7.5	ETFA	50%
7	Selectfluor^®^	1:7.5	CH_3_CN	19%
8	Selectfluor^®^	1:7.5	toluene	10%
9	*m*-CPBA	1:7.5	CHCl_3_	50%
10	gr. Oxone^®^	1:7.5	CHCl_3_	<5%
11	*N*-pyridine oxide	1:7.5	CHCl_3_	<5%
12^d^	Selectfluor^®^	1:7.5	CHCl_3_	64%
13^e^	Selectfluor^®^	1:7.5	CHCl_3_	<5%
14	–	1:7.5	CHCl_3_	<5%
15	Selectfluor^®^	–	CHCl_3_	<5%

^a^Standard reaction conditions: alkyne **1** (0.2 mmol), *p*-TolI (20 mol %), oxidant (1.5 equiv), solvent (0.5 mL), amine/HF source (0.5 mL), ambient temperature, 24 h. ^b^See Supporting Information File 1 for the exact calculation of the amine/HF mixtures. ^c^Determined by ^19^F NMR analysis of the crude reaction mixture using ethyl fluoroacetate as the internal standard. ^d^Reaction was performed at 50 °C. ^e^Control reaction without catalyst.

Having established that an amine/HF ratio of 1:7.5 provides the optimal Brønsted acidity for catalysis, a solvent screen was conducted to assess the effect of the reaction medium. Chlorinated solvents proved to be most effective, with reactions performed in CHCl_3_ slightly outperforming those in CH_2_Cl_2_ (64% and 56% yield, respectively, Table 1, entries 2 and 4). Fluorinated solvents such as hexafluoroisopropanol (HFIP) or ethyl trifluoroacetate (ETFA) led to similar results (52% and 50% yield, respectively, Table 1, entries 5 and 6). Switching to non-halogenated solvents such as acetonitrile or toluene proved to be detrimental (19% and 10%, respectively, Table 1, entries 7 and 8). A screen of common oxidants for I(I)/I(III) catalysis confirmed Selectfluor^®^ as being ideally suited, in line with our previous observations [26–30]. Whereas *m*-CPBA generated the desired α-fluoroketone **2** (50% yield), ground Oxone^®^ or *N*-pyridine oxide proved to be ineffective (Table 1, entries 9–11). Increasing the reaction temperature to 50 °C led to the same outcome as at ambient temperature (64% yield, Table 1, entry 12). Finally, the control reactions in the absence of catalyst, oxidant and HF source were performed and supported the involvement of the postulated I(I)/I(III) cycle (Table 1, entries 13–15).

Since the conditions described in Table 1, entry 2 furnished the desired α-fluoroketone **2** in a respectable yield without the addition of H_2_O, efforts to establish the influence of rigorous exclusion and excess addition were conducted (Table 2). In both scenarios, comparable results were observed (without H_2_O 64%, with addition of 1.0 equiv 64% yield, Table 2, entries 1 and 2). Further increasing the amount of H_2_O from 2.0 equiv to 10 equiv suppressed reactivity (to 29% yield, Table 2, entries 3–5). In addition, a nearly linear correlation between the equivalents of added H_2_O and the product yield was observed (Table 2 insert). Cognisant that H_2_O is likely being introduced to the reaction via the HF source, and to prevent reproducibility issues due to variation of the H_2_O content, 1.0 equiv was added in the general procedure to enable comparison.

**Table 2:** Investigating the role of H_2_O during the reaction_._^a^

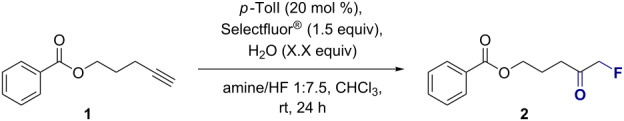			

entry	equiv of H_2_O	yield^b^	correlation of equiv H_2_O versus yield^c^

1	–	64%	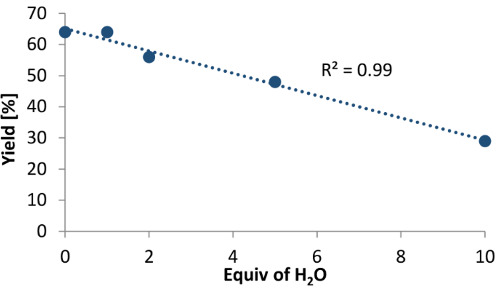
**2**	**1.0**	**64%**	
3	2.0	56%	
4	5.0	48%	
5	10	29%	

^a^Standard reaction conditions: alkyne **1** (0.2 mmol), *p*-TolI (20 mol %), Selectfluor^®^ (1.5 equiv), H_2_O (0–10 equiv), CHCl_3_ (0.5 mL), amine/HF 1:7.5 (0.5 mL), ambient temperature, 24 h. See Supporting Information File 1 for the exact calculation of the amine/HF mixtures. ^b^Determined by ^19^F NMR analysis of the crude reaction mixture using ethyl fluoroacetate as internal standard. ^c^Linear correlation of the equivalent of H_2_O and the corresponding yield of **2**.

**Scheme 1:**
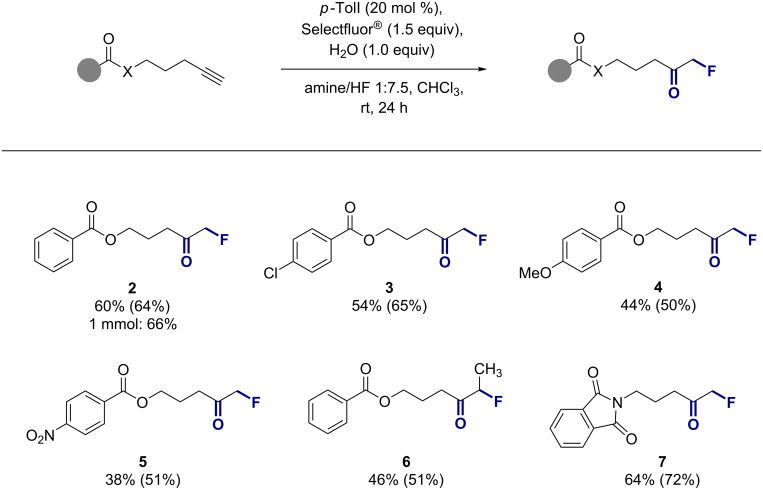
Substrate scope with standard reaction conditions: alkyne (0.2 mmol), *p*-TolI (20 mol %), Selectfluor^®^ (1.5 equiv), H_2_O (1.0 equiv), CHCl_3_ (0.5 mL), amine/HF 1:7.5 (0.5 mL), ambient temperature, 24 h. Yields refer to isolated products while ^19^F NMR yields are given in parentheses (determined by ^19^F NMR analysis of the crude reaction mixture using ethyl fluoroacetate as internal standard). See Supporting Information File 1 for the exact calculation of the amine/HF mixtures.

With the optimised reaction conditions in hand (Table 2, entry 2), the scope and limitations of the title transformation were explored. Initially, the influence of modifying the aryl fragment of the 4-pentynyl benzoate motif was established (Scheme 1). Exposing the standard substrate **1** to the general reaction conditions furnished the desired product **2** in a synthetically useful isolated yield of 60%. Introduction of *para*-substituents was tolerated enabling formation of the chloro, methoxy, and nitro derivatives **3**–**5** with up to 54% yield. The exposure of a model internal alkyne to the standard conditions proved also successful affording **6** in 46% yield. Finally, replacement of the benzoate unit by a phthalimide moiety was possible and led to the protected amine derivative **7** (64% yield).

**Figure 2:**
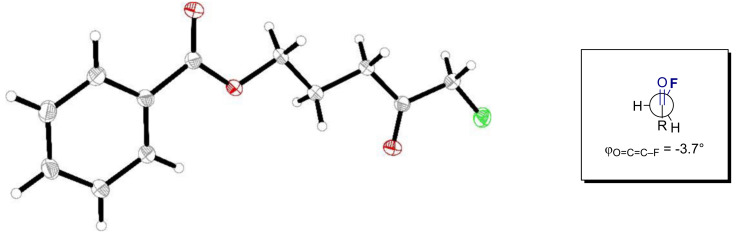
X-ray molecular structure of compound **2**. Conformation of the carbonyl group and the fluoride with a torsion angle of *φ*_O=C-C-F_ = −3.7°. CCDC number 2000136.

The structure of α-fluoroketone **2** was unequivocally established by single crystal X-ray diffraction (Figure 2). Interestingly, a study by Pattison has established that α-fluoroketones preferentially adopt a *cis*-conformation in polar solvents [54]. In the solid state, a dihedral angle of *φ* = −3.7° was observed thereby placing the C–F bond in the same plane as the carbonyl group.

**Figure 3:**
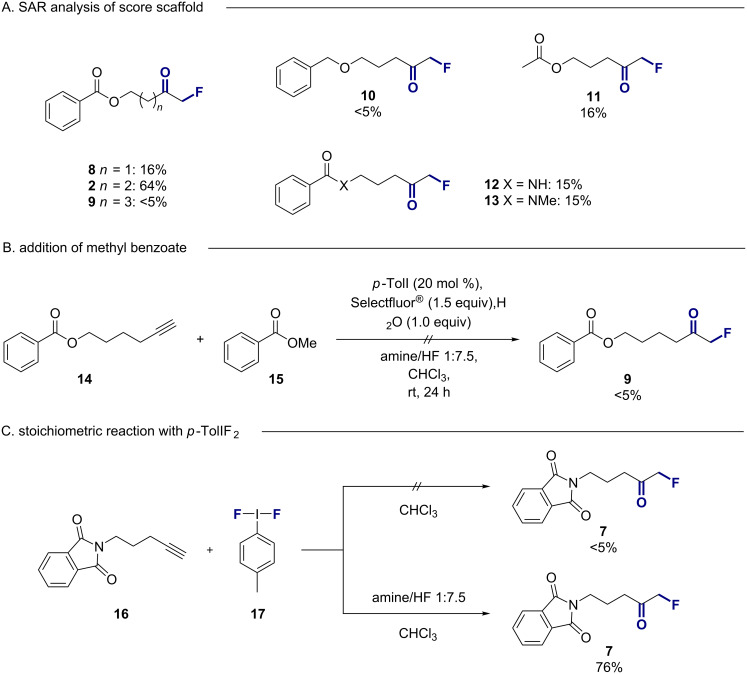
(A) Structure activity relationship of the core scaffold. (B) Exploring the effect of methyl benzoate as an activator. (C) Stoichiometric reaction with *p*-TolIF_2_****17** and alkyne **16**. Yields refer to spectroscopical yields determined by ^19^F NMR analysis of the crude reaction mixtures using ethyl fluoroacetate as an internal standard.

To establish the limitations of the title transformation, a process of substrate editing was initiated (Figure 3). However, efforts to contract or expand the alkynyl chain to four or six carbons, respectively, had a negative impact on the yield of the α*-*fluoroketone (16%, <5% for **8** and **9**, respectively, Figure 3A). Deletion of the ester carbonyl (**10**) and replacement of the phenyl group by methyl (**11**) was also detrimental. From these data it is tempting to speculate upon the involvement of a chair-like transition state in which the intramolecular interaction of the phenyl π-system, carbonyl and alkynyl group are optimally preorganised with a C5 linker. With this in mind, the amide derivatives **12** and **13** were exposed to the standard reaction conditions. Curiously, the reactions were extremely ineffective yielding the respective α-fluoroketones in only 15% yield as determined by ^19^F NMR spectroscopy. This contrasts sharply with the phthalimide derivative **7** (64%). Doping a representative reaction (**14 → 9**) with methyl benzoate (**15**) (Figure 3B) to explore for potential activator effects also proved unsuccessful.

Finally, a stoichiometric reaction of alkynyl phthalimide **16** with freshly prepared *p*-TolIF_2_ **17** demonstrated the importance of the exogenous amine·HF as a Brønsted acid activator (Figure 3C). Similar yields were observed when comparing the stoichiometric reaction and the catalytic process using an amine/HF ratio of 1:7.5.

**Figure 4:**
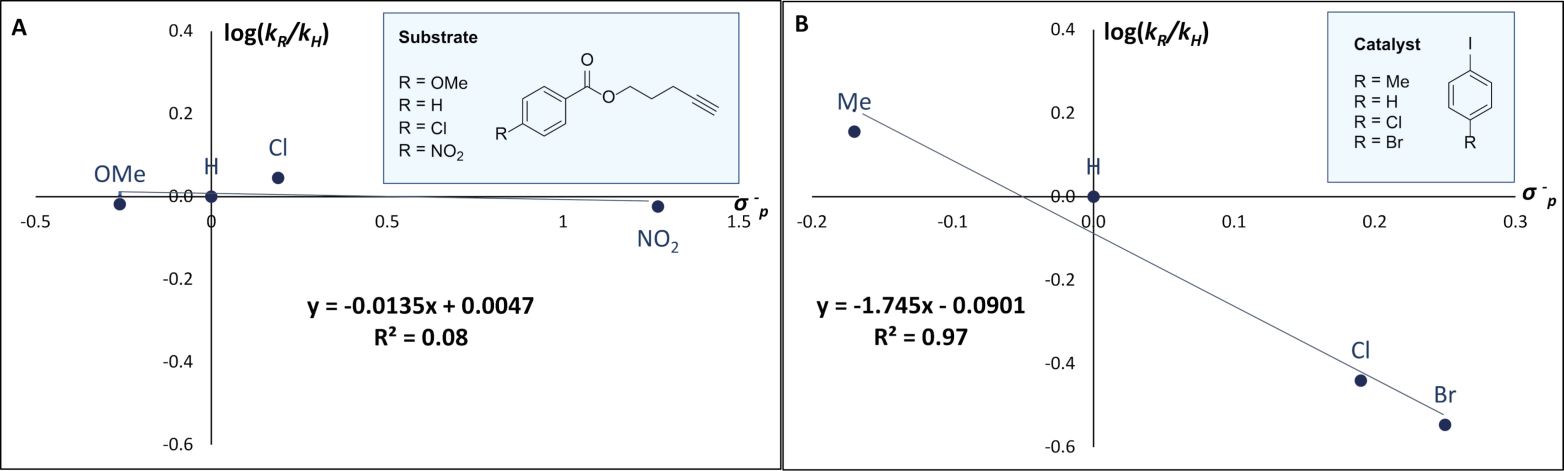
(A) Hammett plot varying the *para*-substitution on the alkyne (*ρ* ≈ 0). (B) Hammett plot varying the *para*-substitution on the catalyst (*ρ* < 0).

To interrogate the importance of the benzoate group in the substrate, a Hammett analysis was conducted. To that end, log(*k*_R_/*k*_H_) was plotted against the Hammett *σ*^−^_p_ parameter for the *para*-substituted pentynyl benzoates with R = OMe, H, Cl, and NO_2_ (Figure 4A). However, the resulting slope of the Hammett plot was essentially zero (*ρ* ≈ 0). In contrast, a second Hammett analysis in which the substitution of the iodoarene catalyst was explored (R = Me, H, Cl, and Br) revealed a build-up of positive charge (*ρ* < 0) during the rate-determining step (Figure 4B).

**Figure 5:**
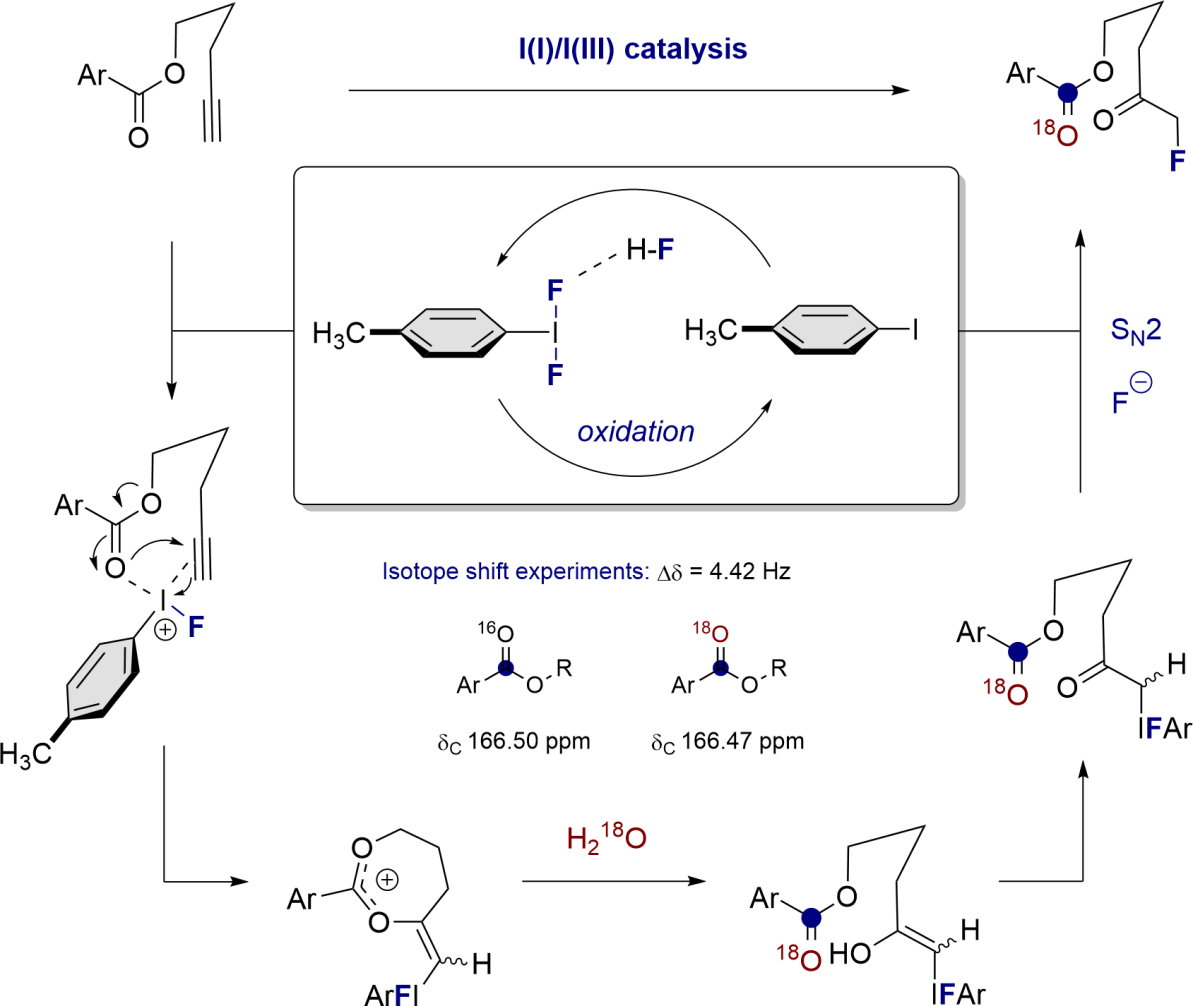
An overview of the I(I)/I(III)-catalysed fluorohydration of alkynes.

From a methodological perspective, this study provides a conceptual platform to unmask the valuable α-fluoroketone motif from alkynes. Selectfluor^®^-mediated oxidation of *p*-TolI in the presence of an amine/HF source, which plays a dual function as fluoride source and Brønsted acid activator, enables in situ generation of *p*-TolIF_2_ (Figure 5). From these preliminary mechanistic investigations, conclusions regarding the extreme sensitivity of the fluorohydration towards changes in the substrate must be drawn with caution. The importance of the pentynyl linker, together with the requirement for the benzoyl ester, suggest a highly preorganised structure [55] in which the carbonyl group functions as a tempered Lewis base upon ligand exchange (Figure 5) [56]. This working hypothesis may also rationalise the deletion experiment (**10**), the recalcitrance of acetyl derivatives, and the striking reactivity disparity between amides (**12**/**13**) and the phthalimide derivative **16**. Finally, to investigate the fate of water in the transformation, the catalytic process was repeated in the presence of H_2_^18^O. Through isotope-shift experiments (please see Supporting Information File 1), it was possible to establish that the aryl ketone of the product contains the ^18^O atom (Δδ_C_ = 4.42 Hz) [57–59]. Formation of the corresponding enol [60], tautomerisation and displacement liberates the α-fluoroketone and regenerates the ArI catalyst.

## Conclusion

In conclusion, the synthesis of α-fluoroketones from 4-pentynyl benzoates based on an I(I)/I(III) catalysis manifold is disclosed. Preliminary mechanistic experiments, supported by Hammett analyses, have identified key determinants that are essential for reaction efficiency. X-ray crystal structure analysis of ketone **2** reveals a dihedral angle of φ_O=C-C-F_ = −3.7°. Although the origin of this specificity requires clarification, this fluorohydration reaction constitutes a rare case of substrate specificity in small-molecule catalysis. It is envisaged that this organocatalytic variant of the venerable Kucherov reaction will find application in contemporary organofluorine chemistry [61] and contributes to the current interest in alkyne functionalisation via I(I)/I(III) catalysis [62–63].

## Supporting Information

File 1 Experimental details, characterization data and copies of spectra.
