# Supplementary material for: Fluorohydration of alkynes via I(I)/I(III) catalysis
Source: Beilstein J Org Chem. 2020 Jul 10;16:1627–35. doi: 10.3762/bjoc.16.135 (PMC7356369; doi:10.3762/bjoc.16.135)
Supplement: File 1 — Experimental details, characterization data and copies of spectra. [file Beilstein_J_Org_Chem-16-1627-s001.pdf]

BEILSTEIN JOURNAL OF ORGANIC CHEMISTRY

# Supporting Information

for

# Fluorohydration of alkynes via I(I)/I(III) catalysis

Jessica Neufeld, Constantin G. Daniliuc and Ryan Gilmour

*Beilstein J. Org. Chem.* **2020,** *16,* 1627–1635. doi:10.3762/bjoc.16.135

# Experimental details, characterization data and copies of spectra

License and Terms: This is a supporting information file under the terms of the Creative Commons Attribution License (http://creativecommons.org/licenses/by/4.0). Please note that the reuse, redistribution and reproduction in particular requires that the authors and source are credited.
The license is subject to the *Beilstein Journal of Organic Chemistry* terms and conditions: (https://www.beilstein-journals.org/bjoc)

# Table of contents


I. General information........................................................... S2

II. Optimisation............................................................... S4

III. Synthesis of starting materials........................................... S7

IV. Synthesis of α-fluoroketones................................................ S16

V. SAR analysis of score scaffold.............................................. S22

VI. Mechanistic and kinetic studies............................................ S24

VII. X-ray crystallographic data................................................ S35

VIII. NMR spectra of unreported starting materials.............................. S37

IX. NMR spectra of α-fluoroketones.............................................. S38

X. References.................................................................. S50

# I. General Information

All chemicals were purchased as reagent grade and used as received unless otherwise stated. Solvents for extraction or purification were purchased as technical grade and distilled on the rotary evaporator prior to use (exception: $Et_2O$ was used as purchased). Dry solvents were dried by a Grubbs-type purification system including columns packed with molecular sieves and aluminium oxide. Column chromatography was performed using silica gel (40–63 µm; VWR Chemicals) as stationary phase. Reaction monitoring was achieved by analytical thin layer chromatography (TLC) on aluminium foils pre-coated with silica gel 60 $F_{254}$ (Merck). Compounds were visualised with UV light (254 nm) or by chemical staining using a solution of $KMnO_4$ ($KMnO_4$ (10 g), $K_2CO_3$ (65 g), NaOH (1 N, 15 mL) in water (1 L)) followed by heating. Concentration in vacuo was performed at ≈ 10 mbar at 40 °C unless otherwise stated. NMR spectra were measured by the NMR service in the Organisch-Chemisches Institut, Westfälische Wilhelms-Universität Münster on a Bruker Avance II 300, a Bruker Avance II 400, an Agilent DD2 500, or an Agilent DD2 600 spectrometer. Chemical shifts are given relative to TMS and are referenced to the residual solvent peak as internal standard. $^{1}$H NMR spectra are reported as follows: chemical shift δ in ppm (multiplicity, coupling constant $J_{FH}$ and $J_{HH}$ in Hz, number of protons, assignment of proton). $^{13}$C NMR spectra are reported as follows: chemical shift δ in ppm (multiplicity, coupling constant $J_{FC}$ in Hz, number of carbons, assignment of carbon). $^{19}$F NMR spectra are reported as follows: chemical shift δ in ppm (multiplicity, coupling constant $J_{FH}$ in Hz, number of fluorines, assignment of fluorine). The resonance multiplicity is abbreviated as s (singlet), d (doublet), t (triplet), q (quartet) or m (multiplet). Assignments of unknown compounds are based on COSY, HSQC and HMBC spectra. Melting points were measured on a Büchi B-545 melting point apparatus in open

capillaries and are uncorrected. IR spectra were recorded on a Perkin-Elmer 100 FT-IR spectrometer. Absorption bands are reported in wave numbers $\tilde{\upsilon}$ ($cm^{-1}$) and the intensities are reported as w (weak), m (medium), s (strong). Mass spectra were measured by the MS service of the Organisch-Chemisches Institut, Westfälische Wilhelms-Universität Münster on a Bruker Daltonics MicroTof (HRMS-ESI), a Triplequad TSQ 7000 (MS-EI), Triplequad Quattro Micro GC (GC-EI-MS), a Qp5050 Single Quad (GC-EI-MS) or a LTQ Orbitap LTQ XL (HRMS-APCI).

**Amine/HF mixtures**

Amine/HF stock solutions were prepared as follows:

$NEt_3$/HF = amine/HF 1:3

pyr·HF (Olah's reagent) = amine/HF 1:9.23

A mixture of amine/HF 1:4.5 was obtained by mixing 0.159 mL of pyr·HF 1:9.23 and 0.341 mL of $NEt_3$/HF 1:3.

A mixture of amine/HF 1:7.5 was obtained by mixing 0.402 mL of pyr·HF 1:9.23 and 0.098 mL of $NEt_3$/HF 1:3.

# II. Optimisation

### General procedure A for the optimisation

Alkyne **1** (38 mg, 0.2 mmol, 1.0 equiv) and *p*-TolI (8.7 mg, 40 μmol, 20 mol %) were dissolved in the stated solvent (0.5 mL) in a Teflon® screw cap vial. The amine/HF mixture (0.5 mL) and the stated oxidant (0.3 mmol, 1.5 equiv) were added and the reaction mixture was stirred for 24 h at ambient temperature. The reaction mixture was poured into a saturated solution of $NaHCO_3$ and the aqueous layer was extracted with $CH_2Cl_2$ (3×). The combined organic layers were dried over $MgSO_4$, filtered and the solvent was removed in vacuo. The yield was determined by $^{19}F$ NMR analysis of the crude reaction mixture using ethyl fluoroacetate as internal standard.

**Table S1:** Reaction optimisation for the synthesis of α-fluoroketone **2** from alkyne **1**.

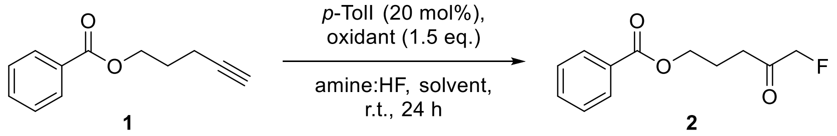


| entry | oxidant | amine/HF | solvent | yield |
|---|---|---|---|---|
| 1 | Selectfluor® | 1:4.5 | $CHCl_3$ | 9% |
| **2** | **Selectfluor®** | **1:7.5** | **$CHCl_3$** | **64%** |
| 3 | Selectfluor® | 1:9.23 | $CHCl_3$ | 45% |
| 4 | Selectfluor® | 1:7.5 | $CH_2Cl_2$ | 56% |
| 5 | Selectfluor® | 1:7.5 | HFIP | 52% |
| 6 | Selectfluor® | 1:7.5 | ETFA | 50% |
| 7 | Selectfluor® | 1:7.5 | $CH_3CN$ | 19% |
| 8 | Selectfluor® | 1:7.5 | toluene | 10% |
| 9 | *m*-CPBA | 1:7.5 | $CHCl_3$ | 50% |
| 10 | gr. Oxone® | 1:7.5 | $CHCl_3$ | <5% |
| 11 | *N*-pyridine oxide | 1:7.5 | $CHCl_3$ | <5% |
| 12[a] | Selectfluor® | 1:7.5 | $CHCl_3$ | 64% |
| 13[b] | Selectfluor® | 1:7.5 | $CHCl_3$ | <5% |
| 14 | - | 1:7.5 | $CHCl_3$ | <5% |
| 15 | Selectfluor® | - | $CHCl_3$ | <5% |

[a] Reaction was performed at 50 °C. [b] Control reaction without catalyst.

**General procedure B for the investigation of the role of $H_2O$ during the reaction**

Alkyne **1** (38 mg, 0.2 mmol, 1.0 equiv) and *p*-TolI (8.7 mg, 40 μmol, 20 mol%) were dissolved in $CHCl_3$ (0.5 mL) in a Teflon® screw cap vial. $H_2O$ (1.0–10 equiv), amine/HF 1:7.5 (0.5 mL), and Selectfluor® (106 mg, 0.3 mmol, 1.5 equiv) were added and the reaction mixture was stirred for 24 h at ambient temperature. The reaction mixture was poured into a saturated solution of $NaHCO_3$ and the aqueous layer was extracted with $CH_2Cl_2$ (3×). The combined organic layers were dried over $MgSO_4$, filtered and the solvent was removed in vacuo. The yield was determined by $^{19}F$ NMR analysis of the crude reaction mixture using ethyl fluoroacetate as internal standard.

**Table S2:** Investigating the role of $H_2O$ during the reaction.

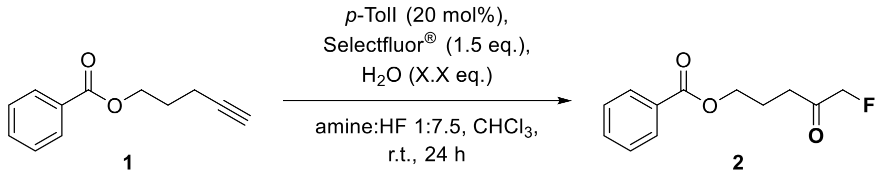


| entry | equiv of $H_2O$ | yield | correlation of equiv $H_2O$ versus yield |
|---|---|---|---|
| 1 | 1.0 | 64% | 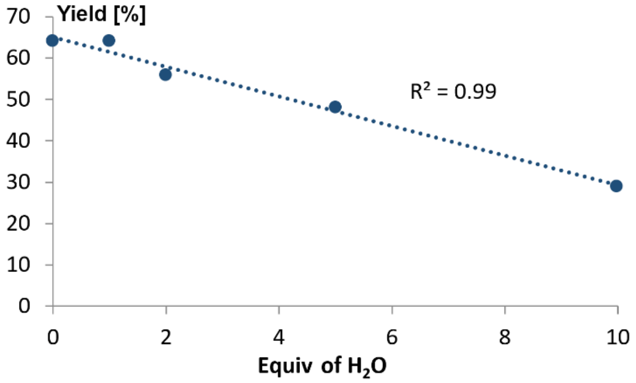 |
| 2 | 2.0 | 56% | |
| 3 | 5.0 | 48% | |
| 4 | 10 | 29% | |

# III. Synthesis of starting materials

**General procedure C for the esterfication of acetyl chlorides with alkynols**

The corresponding alkynol (1.0 equiv) and acetyl chloride (1.1-2.0 equiv) were dissolved in $Et_2O$, $NEt_3$ (1.1–2.0 equiv) was added slowly, and the reaction mixture was stirred for the indicated time at ambient temperature. The reaction mixture was diluted with EtOAc and $H_2O$, and the aqueous layer was extracted with EtOAc (3×). The combined organic layers were washed with $H_2O$ and brine, dried over $MgSO_4$ and filtered. The solvent was removed in vacuo and the crude mixture was purified by column chromatography.

**Pent-4-yn-1-yl benzoate (1)**

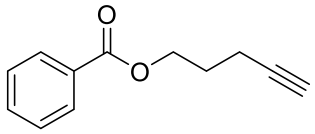


According to **general procedure C**, pent-4-yl-1-ol (0.93 mL, 10 mmol, 1.0 equiv) and benzoyl chloride (1.3 mL, 11 mmol, 1.1 equiv) were dissolved in $Et_2O$ (15 mL), $NEt_3$ (1.5 mL, 11 mmol, 1.1 equiv) was added slowly and the reaction mixture was stirred for 24 h. Column chromatography purification (cyclohexane/EtOAc 98:2) afforded product **1** as colourless oil (1.3 g, 7.3 mmol, 73%).

**R$_f$** (cyclohexane/EtOAc 10:1) = 0.45.

**$^1$H NMR** (400 MHz, $CDCl_3$, 299 K) $\delta$ [ppm] = 8.08-8.00 (m, 2H), 7.59-7.53 (m, 1H), 7.48-7.40 (m, 2H), 4.43 (t, $J_{HH}$ = 6.3 Hz, 2H), 2.39 (td, $J_{HH}$ = 7.0, 2.7 Hz, 2H), 2.06-1.93 (m, 3H).

**MS** (EI) *m/z* $[M]^+$, calcd. for $C_{12}H_{12}O_2^+$: 188.08, found: 188.13.

Analytic data in agreement with the literature [1-2].

**Pent-4-yn-1-yl 4-chlorobenzoate (S1)**

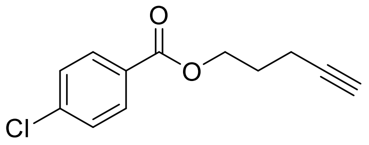

According to **general procedure C**, pent-4-yl-1-ol (0.93 mL, 10 mmol, 1.0 equiv) and 4-chlorobenzoyl chloride (1.4 mL, 11 mmol, 1.1 equiv) were dissolved in $Et_2O$ (25 mL), $NEt_3$ (2.1 mL, 15 mmol, 1.5 equiv) was added slowly, and the reaction mixture was stirred for 16 h. Column chromatography purification (cyclohexane/EtOAc 98:2) afforded product **S1** as white solid (2.1 g, 9.6 mmol, 96%).

$\mathbf{R_f}$ (cyclohexane/EtOAc 10:1) = 0.47.

**M.p.** 26-27 °C.

**$^1$H NMR** (300 MHz, $CDCl_3$, 299 K) $\delta$ [ppm] = 8.04-7.89 (m, 2H), 7.46-7.37 (m, 2H), 4.42 (t, $J_{HH}$ = 6.2 Hz, 2H), 2.38 (td, $J_{HH}$ = 7.0, 2.7 Hz, 2H), 2.07-1.89 (m, 3H).

**HRMS** (ESI) *m/z* $[M+Na]^+$, calcd. for $C_{12}H_{11}O_2ClNa^+$: 245.0340, found: 245.0340.

Analytic data in agreement with the literature [3].

**Pent-4-yn-1-yl 4-methoxybenzoate (S2)**

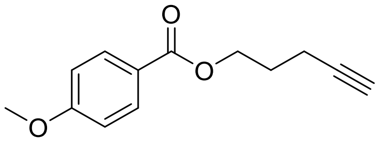

According to **general procedure C**, pent-4-yl-1-ol (0.47 mL, 5.0 mmol, 1.0 equiv) and 4-methoxy benzoyl chloride (1.0 mL, 7.5 mmol, 1.5 equiv) were dissolved in $Et_2O$ (15 mL), $NEt_3$ (1.0 mL, 7.5 mmol, 1.5 equiv) was added slowly, and the reaction mixture was stirred for 24 h. Column chromatography purification (cyclohexane/EtOAc 20:1) afforded product **S2** as white solid (0.67 g, 3.1 mmol, 61%).

$\mathbf{R_f}$ (cyclohexane/EtOAc 10:1) = 0.24.

**M.p.** 32-33 °C.

**$^1$H NMR** (300 MHz, $CDCl_3$, 299 K) $\delta$ [ppm] = 8.10-7.95 (m, 2H), 6.99-6.73 (m, 2H), 4.40 (t, $J_{HH}$ = 6.2 Hz, 2H), 3.86 (s, 3H), 2.38 (td, $J_{HH}$ = 7.1, 2.7 Hz, 2H), 2.09-1.87 (m, 3H).

**HRMS** (ESI) *m/z* $[M+Na]^+$, calcd. for $C_{13}H_{14}O_3Na^+$: 241.0835, found: 241.0834.

Analytic data in agreement with the literature [2].

**Pent-4-yn-1-yl 4-nitrobenzoate (S3)**

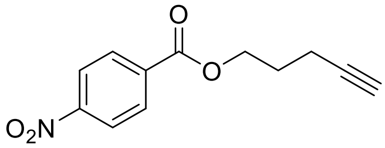


According to **general procedure C**, pent-4-yl-1-ol (0.93 mL, 10 mmol, 1.0 equiv) and 4-nitrobenzoyl chloride (2.8 g, 15 mmol, 1.5 equiv) were dissolved in $Et_2O$ (25 mL), $NEt_3$ (2.1 mL, 15 mmol, 1.5 equiv) was added slowly, and the reaction mixture was stirred for 16 h. Column chromatography purification (cyclohexane/EtOAc 20:1) afforded product **S3** as pale yellow solid (1.5 g, 6.6 mmol, 66%).

$\mathbf{R_f}$ (cyclohexane/EtOAc 10:1) = 0.26.

**M.p.** 43-44 °C.

**$^1$H NMR** (300 MHz, $CDCl_3$, 299 K) $\delta$ [ppm] = 8.32-8.26 (m, 2H), 8.25-8.18 (m, 2H), 4.50 (t, $J_{HH}$ = 6.3 Hz, 2H), 2.40 (td, $J_{HH}$ = 7.0, 2.7 Hz, 2H), 2.10-1.92 (m, 3H).

**HRMS** (ESI) *m/z* $[M+Na]^+$, calcd. for $C_{12}H_{11}NO_4Na^+$: 256.0580, found: 256.0580.

Analytic data in agreement with the literature [4].

**Hex-4-yn-1-ol (S4)**

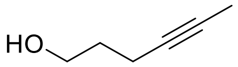


According to a modified procedure [5], KO*t*-Bu (3.4 g, 30 mmol, 2.0 equiv) was added to a solution of hex-5-yn-1-ol (1.7 mL, 15 mmol, 1.0 equiv) in DMSO (45 mL) under argon atmosphere, and the reaction mixture was stirred for 12 h at ambient temperature. Aqueous HCl (2 M) was added and the aqueous layer was extracted with $Et_2O$ (3×). The combined organic layers were dried over $MgSO_4$, filtered, and the solvent removed in vacuo. Column chromatography purification (*n*-pentane/$Et_2O$ 3:1) afforded product **S4** as colourless oil (0.92 g, 9.3 mmol, 62%).

$\mathbf{R_f}$ (*n*-pentane/$Et_2O$ 3:1) = 0.10.

**$^1$H NMR** (300 MHz, $CDCl_3$, 299 K) $\delta$ [ppm] = 3.74 (t, $J_{HH}$ = 6.1 Hz, 2H), 2.24 (tt, $J_{HH}$ = 7.0, 2.6 Hz, 2H), 1.82-1.63 (m, 6H).

**MS** (EI) *m/z* $[M]^+$, calcd. for $C_6H_{10}O^+$: 98.07, found: 98.13.

Analytic data in agreement with the literature [5].

**Hex-4-yn-1-yl benzoate (S5)**

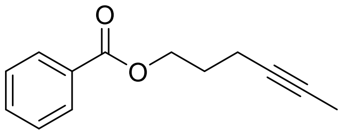

According to **general procedure C**, hex-4-yl-1-ol (**S4**) (0.30 g, 3.0 mmol, 1.0 equiv) and benzoyl chloride (0.38 mL, 3.3 mmol, 1.1 equiv) were dissolved in $Et_2O$ (5 mL), $NEt_3$ (0.46 mL, 3.3 mmol, 1.1 equiv) was added slowly, and the reaction mixture was stirred for 16 h. Column chromatography purification (cyclohexane/EtOAc 98:2) afforded product **S5** as colourless oil (0.47 g, 2.3 mmol, 77%).

$\mathbf{R_f}$ (cyclohexane/EtOAc 10:1) = 0.42.

**$^1$H NMR** (400 MHz, $CDCl_3$, 299 K) $\delta$ [ppm] = 8.09-7.97 (m, 2H), 7.59-7.51 (m, 1H), 7.49-7.37 (m, 2H), 4.41 (t, $J_{HH}$ = 6.3 Hz, 2H), 2.33 (tq, $J_{HH}$ = 7.1, 2.5 Hz, 2H), 1.95 (tt, $J_{HH}$ = 6.7, 6.7 Hz, 2H), 1.77 (t, $J_{HH}$ = 2.6 Hz, 3H).

**MS** (EI) *m/z* $[M]^+$, calcd. for $C_{13}H_{14}O_2^+$: 202.10, found: 202.15.

Analytic data in agreement with the literature [6].

**But-3-yn-1-yl benzoate (S6)**

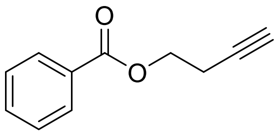

According to **general procedure C**, but-3-yl-1-ol (0.38 mL, 5.0 mmol, 1.0 equiv) and benzoyl chloride (0.87 mL, 7.5 mmol, 1.5 equiv) were dissolved in $Et_2O$ (15 mL), $NEt_3$ (1.0 mL, 7.5 mmol, 1.5 equiv) was added slowly, and the reaction mixture was stirred for 15 h. Column chromatography purification (cyclohexane/EtOAc 98:2) afforded product **S6** as colourless oil (0.40 g, 2.3 mmol, 45%).

$\mathbf{R_f}$ (cyclohexane/EtOAc 10:1) = 0.41.

**$^1$H NMR** (300 MHz, $CDCl_3$, 299 K) $\delta$ [ppm] = 8.10-8.01 (m, 2H), 7.61-7.50 (m, 1H), 7.49-7.39 (m, 2H), 4.43 (t, $J_{HH}$ = 6.8 Hz, 2H), 2.68 (td, $J_{HH}$ = 6.8, 2.7 Hz, 2H), 2.03 (t, $J_{HH}$ = 2.6 Hz, 1H).

**MS** (EI) *m/z* $[M]^+$, calcd. for $C_{11}H_{10}O_2^+$: 174.07, found: 174.13.

Analytic data in agreement with the literature [7].

**Hex-5-yn-1-yl benzoate (14)**

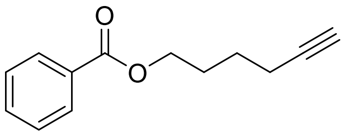

According to **general procedure C**, hex-5-yl-1-ol (1.8 mL, 15 mmol, 1.0 equiv) and benzoyl chloride (2.6 mL, 23 mmol, 1.5 equiv) were dissolved in $Et_2O$ (15 mL), $NEt_3$ (3.1 mL, 15 mmol, 1.5 equiv) was added slowly, and the reaction mixture was stirred for 24 h. Column chromatography purification (cyclohexane/EtOAc 98:2) afforded product **14** as colourless oil (2.0 g, 9.7 mmol, 65%).

$\mathbf{R_f}$ (cyclohexane/EtOAc 10:1) = 0.38.

**$^1$H NMR** (300 MHz, $CDCl_3$, 299 K) $\delta$ [ppm] = 8.09-7.99 (m, 2H), 7.62-7.50 (m, 1H), 7.50-7.38 (m, 2H), 4.35 (t, $J_{HH}$ = 6.4 Hz, 2H), 2.29 (td, $J_{HH}$ = 7.0, 2.6 Hz, 2H), 2.03-1.78 (m, 3H), 1.79-1.59 (m, 2H).

**HRMS** (ESI) *m/z* $[M+Na]^+$, calcd. for $C_{13}H_{14}NaO_2^+$: 225.0886, found: 225.0884.

Analytic data in agreement with the literature [8].

**(Pent-4-yn-1-yloxy)methyl)benzene (S7)**

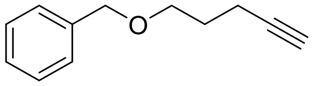

According to a modified procedure [9], pent-4-yl-1-ol (0.93 mL, 10 mmol, 1.0 equiv) and imidazole (14 mg, 0.2 mmol, 2 mol %) were dissolved in dry THF (10 mL) under an argon atmosphere. Sodium hydride (60% suspension in mineral oil, 1.2 g, 30 mmol, 3.0 equiv) was added at 0 °C and the reaction mixture was stirred for 30 min at this temperature. Then, benzyl bromide (1.4 mL, 12 mmol, 1.2 equiv) was added at 0 °C and the reaction mixture was stirred for 22 h at ambient temperature. The reaction mixture was diluted with $H_2O$ and EtOAc and the organic layer was washed with $H_2O$ (2×), a saturated solution of $NH_4Cl$ (2×) and brine (1×). The combined organic layers were dried over $MgSO_4$, filtered, and the solvent was removed in vacuo. Column chromatography purification

($n$-pentane/$CH_2Cl_2$ 10:1 → 1:1) afforded product **S7** as colourless oil (1.6 g, 9.2 mmol, 92%).

**$R_f$** ($n$-pentane/$CH_2Cl_2$ 10:1) = 0.10.

**$^1$H NMR** (300 MHz, $CDCl_3$, 299 K) $\delta$ [ppm] = 7.40-7.27 (m, 5H), 4.53 (s, 2H), 3.59 (t, $J_{HH}$ = 6.1 Hz, 2H), 2.33 (td, $J_{HH}$ = 7.1, 2.7 Hz, 2H), 1.95 (t, $J_{HH}$ = 2.7 Hz, 1H), 1.85 (tt, $J_{HH}$ = 6.6, 6.6 Hz, 2H).

**MS** (EI) *m/z* $[M]^+$, calcd. for $C_{12}H_{14}O^+$: 174.10, found: 174.17.

Analytic data in agreement with the literature [10].

**Pent-4-yn-1-yl acetate (S8)**

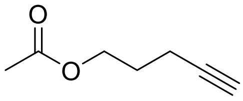


According to **general procedure C**, pent-4-yl-1-ol (0.32 mL, 3.5 mmol, 1.0 equiv) and acetyl chloride (0.28 mL, 3.9 mmol, 1.1 equiv) were dissolved in $Et_2O$ (5 mL), $NEt_3$ (0.54 mL, 3.9 mmol, 1.1 equiv) was added slowly, and the reaction mixture was stirred for 18 h. Column chromatography purification ($n$-pentane/EtOAc 20:1) afforded product **S8** as colourless oil (0.12 g, 0.92 mmol, 26%).

**$R_f$** ($n$-pentane/EtOac 10:1) = 0.78.

**$^1$H NMR** (400 MHz, $CDCl_3$, 299 K) $\delta$ [ppm] = 4.17 (td, $J_{HH}$ = 6.3, 1.0 Hz, 2H), 2.29 (tdd, $J_{HH}$ = 7.0, 2.7, 0.9 Hz, 2H), 2.05 (d, $J_{HH}$ = 1.0 Hz, 3H), 1.97 (td, $J_{HH}$ = 2.7, 0.9 Hz, 1H), 1.86 (ddd, $J_{HH}$ = 7.3, 6.8, 1.0 Hz, 2H).

**MS** (EI) *m/z* $[M]^+$, calcd. for $C_7H_{10}O_2^+$: 125.07, found: 125.12.

Analytic data in agreement with the literature [11].

**N-(Pent-4-yn-1-yl)benzamide (S9)**

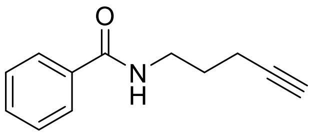

According to a modified procedure [12], 2-(pent-4-yn-1-yl)isoindoline-1,3-dione (**16**) (1.1 g, 5.0 mmol, 1.0 equiv) was dissolved in EtOH (13 mL). $N_2H_4$ (2.5 mL, 50 mmol, 10 equiv) was added to the slurry until it got clear and the reaction mixture was stirred for 3 h at 85 °C. Then, $H_2O$ was added, followed by a solution of HCl (2 M) to adjust a pH of 3. The precipitate was filtered and the filtrate was concentrated in vacuo. The residue was cooled to 0 °C and a solution of NaOH (10 M, 5 mL) was added. The aqueous layer was extracted with $CH_2Cl_2$ (3×) and the combined organic layers were dried over $MgSO_4$, filtered, and concentrated in vacuo. Pent-4-yn-1-amine was used crude without any purification in the next step.

According to **general procedure C**, pent-4-yn-1-amine (1.8 mL, 15 mmol, 1.0 equiv) and benzoyl chloride (0.87 mL, 7.5 mmol, 1.5 equiv) were dissolved in $Et_2O$ (10 mL), $NEt_3$ (1.0 mL, 7.5 mmol, 1.5 equiv) was added slowly, and the reaction mixture was stirred for 15 h. Column chromatography purification (cyclohexane/EtOAc 8:2) afforded product **S9** as white solid (0.43 g, 2.3 mmol, 46% over two steps).

**M.p.** 65-67 °C.

**$R_f$** (cyclohexane/EtOAc 7:3) = 0.26.

**$^1$H NMR** (300 MHz, $CDCl_3$, 299 K) $\delta$ [ppm] = 7.81-7.72 (m, 2H), 7.55-7.37 (m, 3H), 6.41 (s, 1H), 3.60 (q, $J_{HH}$ = 6.6 Hz, 2H), 2.33 (td, $J_{HH}$ = 6.8, 2.7 Hz, 2H), 2.03 (t, $J_{HH}$ = 2.7 Hz, 1H), 1.87 (dd, $J_{HH}$ = 6.6, 6.8 Hz, 2H).

**HRMS** (ESI) *m/z* $[M+Na]^+$, calcd. for $C_{12}H_{13}NONa^+$: 210.0889, found: 210.0885.

Analytic data in agreement with the literature [13].

**N-Methyl-N-(pent-4-yn-1-yl)benzamide (S10)**

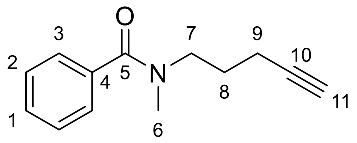


According to a modified procedure [14], *N*-(pent-4-yn-1-yl)benzamide (**S9**, 0.38 g, 2.0 mmol, 1.0 equiv) was dissolved in DMF (10 mL) under an argon atmosphere. Then, NaH (60% in mineral oil, 0.12 g, 3.0 mmol, 1.5 equiv) was added at 0 °C and the reaction mixture was stirred for 30 min before MeI (0.25 mL, 4.0 mmol, 2.0 equiv) was added and the mixture was stirred for another 14 h. A saturated solution of $NH_4Cl$ was added and the aqueous solution was extracted with EtOAc (3×). The combined organic layers were washed with brine, dried over $MgSO_4$, filtered and the solvent was removed in vacuo. Column chromatography purification (cyclohexane/EtOAc 7:3) afforded product **S10** as colourless oil (0.34 g, 1.7 mmol, 84%).

$\mathbf{R_f}$ (cyclohexane/EtOAc 7:3) = 0.19.

$^{1}$**H NMR** (500 MHz, DMSO-$d_6$, 373 K) $\delta$ [ppm] = 7.45-7.39 (m, 3H, H1/H3), 7.39-7.34 (m, 2H, H2), 3.43 (t, $^{3}J_{HH}$ = 7.3 Hz, 2H, H7), 2.93 (s, 3H, H6), 2.52 (t, $^{4}J_{HH}$ = 2.7 Hz, 1H, H11), 2.17 (td, $^{3}J_{HH}$ = 7.1 Hz, $^{4}J_{HH}$ = 2.7 Hz, 2H, H9), 1.76 (tt, $^{3}J_{HH}$ = 7.2, 7.1 Hz, 2H, H8).

$^{13}$**C NMR** (126 MHz, DMSO-$d_6$, 373 K) $\delta$ [ppm] = 169.9 (1C, C5), 136.6 (1C, C4), 128.5 (1C, C1), 127.6 (2C, C3), 126.0 (2C, C2), 83.2 (1C, C10), 70.2 (1C, C11), 47.3 (1C, C7), 25.8 (1C, C8), 14.9 (1C, C9).

**IR** (ATR) $\tilde{\upsilon}$ [cm$^{-1}$] = 3289 (w), 3235 (w), 2933 (w), 1623 (s), 1577 (m), 1499 (m), 1480 (m), 1445 (m), 1431 (m), 1399 (m), 1305 (m), 1260 (w), 1234 (w), 1216 (w), 1149 (w), 1072 (m), 1026 (m), 924 (w), 848 (w), 788 (m), 698 (s).

**HRMS** (ESI) *m/z* [M+Na]$^+$, calcd. for $C_{13}H_{15}NONa^+$: 224.1046, found: 224.1040.

**Difluoro(*p*-tolyl)-$\lambda^3$-iodane (17)**

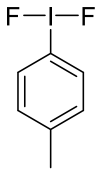

According to a modified procedure [15], *p*-iodotoluene (0.22 g, 1.0 mmol, 1.0 equiv) and Selectfluor® (1.2 g, 3.5 mmol, 3.5 equiv) were dissolved in dry MeCN (25 mL) under an argon atmosphere. The reaction mixture was flushed three times with argon and $NEt_3$/HF 1:3 (0.20 mL) was added. The reaction mixture was wrapped in aluminium foil and stirred for 24 h at ambient temperature. The reaction mixture was then evaporated to dryness and a mixture of $CHCl_3$/*n*-hexane 1:3 (4 mL) was added to the slurry. The organic material was transferred to a Schlenk tube and the process was repeated four times. The solvent was evaporated to dryness at 0 °C to obtain product **17** as yellowish needles (0.16 mg, 0.62 mmol, 62%).

**$^1$H NMR** (300 MHz, $CDCl_3$, 299 K) $\delta$ [ppm] = 7.94-7.71 (m, 2H), 7.44-7.35 (m, $J_{HH}$ = 8.4 Hz, 2H), 2.47 (s, 3H).

**$^{19}$F NMR** (282 MHz, $CDCl_3$, 299 K) $\delta$ [ppm] = -176.8 (s, 2F).

Analytic data in agreement with the literature [15-16].

# IV. Synthesis of α-fluoroketones

**General procedure D for the synthesis of α-fluoroketones**

The alkyne (0.2 mmol, 1.0 equiv) and *p*-TolI (8.7 mg, 40 µmol, 20 mol %) were dissolved in $CHCl_3$ (0.5 mL) in a Teflon® screw cap vial. $H_2O$ (3.6 µL, 0.2 mmol, 1.0 equiv), amine/HF 1:7.5 (0.5 mL), and Selectfluor® (106 mg, 0.3 mmol, 1.5 equiv) were added, and the reaction mixture was stirred for 24 h at ambient temperature. Then, the reaction mixture was poured into a saturated solution of $NaHCO_3$ and the aqueous layer was extracted with $CH_2Cl_2$ (3×). The combined organic layers were dried over $MgSO_4$, filtered and the solvent was removed in vacuo. The yield was determined by $^{19}F$ NMR analysis of the crude reaction mixture using ethyl fluoroacetate as internal standard. Then, the crude reaction mixture was purified by column chromatography affording the desired product.

**5-Fluoro-4-oxopentyl benzoate (2)**

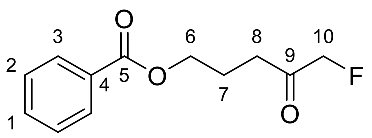


The reaction was performed according to **general procedure D** using pent-4-yn-1-yl benzoate (**1**) (38 mg, 0.2 mmol, 1.0 equiv). Column chromatography purification (cyclohexane/EtOAc 10:1) afforded product **2** as white solid (27 mg, 0.12 mmol, 60%; $^{19}F$ NMR yield: 64%).

1.0 mmol scale:

The reaction was performed according to **general procedure C** using pent-4-yn-1-yl benzoate (**1**) (0.19 mg, 1.0 mmol, 1.0 equiv), *p*-TolI (44 mg, 0.40 mmol, 20 mol %), $H_2O$ (18 µL, 1.0 mmol, 1.0 equiv), Selectfluor® (0.53 g, 1.5 mmol, 1.5 equiv),

$CHCl_3$ (2.5 mL), and amine/HF 1:7.5 (2.5 mL). Column chromatography purification (cyclohexane/EtOAc 9:1) afforded product **2** as white solid (0.15 mg, 0.66 mmol, 66%).

**R$_f$** (cyclohexane/EtOAc 8:2) = 0.29.

**M.p.** 40-41 °C.

**$^1$H NMR** (500 MHz, $CDCl_3$, 299 K) $\delta$ [ppm] = 8.04-7.99 (m, 2H, H3), 7.60-7.52 (m, 1H, H1), 7.48-7.39 (m, 2H, H2), 4.82 (d, $^2J_{FH}$ = 47.6 Hz, 2H, H10), 4.36 (t, $^3J_{HH}$ = 6.3 Hz, 2H, H6), 2.73 (td, $^3J_{HH}$ = 7.1 Hz, $^4J_{FH}$ = 2.4 Hz, 2H, H8), 2.12 (tt, $^3J_{HH}$ = 7.2, 6.8 Hz, 2H, H7).

**$^{13}$C NMR** (126 MHz, $CDCl_3$, 299 K) $\delta$ [ppm] = 206.3 (d, $^2J_{FC}$ = 19.9 Hz, 1C, C9), 166.6 (1C, C5), 133.2 (1C, C1), 130.2 (1C, C4), 129.7 (2C, C3), 128.5 (2C, C2), 85.1 (d, $^1J_{FC}$ = 184.8 Hz, 1C, C10), 64.0 (1C, C6), 34.9 (1C, C8), 22.1 (1C, C7).

**$^{19}$F NMR** (470 MHz, $CDCl_3$, 299 K) $\delta$ [ppm] = -227.8 (tt, $^2J_{FH}$ = 47.6 Hz, $^4J_{FH}$ = 2.5 Hz, 1F, F10).

**IR** (ATR) $\tilde{\nu}$ [cm$^{-1}$] = 2969 (w), 2900 (w), 1711 (s), 1585 (w), 1481 (m), 1453 (m), 1434 (m), 1412 (m), 1399 (m), 1316 (m), 1280 (s), 1180 (m), 1127 (s), 1116 (s), 1079 (m), 1025 (m), 998 (s), 851 (w), 806 (w), 706 (s), 684 (s).

**HRMS** (ESI) *m/z* $[M+Na]^+$, calcd. for $C_{12}H_{13}O_3FNa^+$: 247.0741, found: 247.0741.

**5-Fluoro-4-oxopentyl 4-chlorobenzoate (3)**

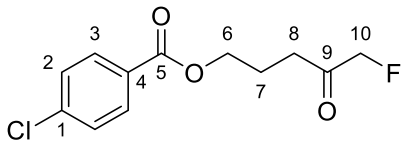


The reaction was performed according to **general procedure D** using pent-4-yn-1-yl 4-chlorobenzoate (**S1**) (45 mg, 0.2 mmol, 1.0 equiv). Column chromatography purification (cyclohexane/EtOAc 10:1) afforded product **3** as white solid (28 mg, 0.11 mmol, 54%; $^{19}$F NMR yield: 65%).

**R$_f$** (cyclohexane/EtOAc 8:2) = 0.30.

**M.p.** 39-40 °C.

**$^{1}$H NMR** (599 MHz, $CDCl_3$, 299 K) $\delta$ [ppm] = 7.97-7.93 (m, 2H, H3), 7.44-7.40 (m, 2H, H2), 4.82 (d, $^{2}J_{FH}$ = 47.7 Hz, 2H, H10), 4.36 (t, $^{3}J_{HH}$ = 6.3 Hz, 2H, H6), 2.73 (td, $^{3}J_{HH}$ = 7.1 Hz, $^{4}J_{FH}$ = 2.5 Hz, 2H, H8), 2.12 (tt, $^{3}J_{HH}$ = 7.1, 6.7 Hz, 2H, H7).

**$^{13}$C NMR** (151 MHz, $CDCl_3$, 299 K) $\delta$ [ppm] = 206.3 (d, $^{2}J_{FC}$ = 20.3 Hz, 1C, C9), 165.8 (1C, C5), 139.7 (1C, C4), 131.1 (2C, C3), 128.9 (2C, C2), 128.7 (1C, C1), 85.2 (d, $^{1}J_{FC}$ = 185.0 Hz, 1C, C10), 64.3 (1C, C6), 35.0 (1C, C8), 22.1 (1C, C7).

**$^{19}$F NMR** (564 MHz, $CDCl_3$, 299 K) $\delta$ [ppm] = -227.8 (tt, $^{2}J_{FH}$ = 47.7 Hz, $^{4}J_{FH}$ = 2.6 Hz, 1F, F10).

**IR** (ATR) $\tilde{\upsilon}$ [cm$^{-1}$] = 2968 (w), 2905 (w), 1730 (m), 1711 (s), 1590 (m), 1478 (m), 1455 (w), 1402 (m), 1278 (s), 1172 (w), 1129 (m), 1117 (m), 1108 (m), 1093 (s), 1018 (m), 993 (s), 965 (m), 854 (s), 842 (m), 757 (s), 719 (m), 683 (m).

**HRMS** (ESI) *m/z* $[M+Na]^+$, calcd. for $C_{12}H_{12}O_3ClFNa^+$: 281.0351, found: 281.0350.

**5-Fluoro-4-oxopentyl 4-methoxybenzoate (4)**

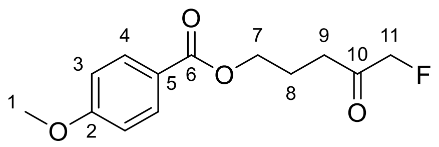


The reaction was performed according to **general procedure D** using pent-4-yn-1-yl 4-methoxybenzoate (**S2**) (44 mg, 0.2 mmol, 1.0 equiv). Column chromatography purification (cyclohexane/EtOAc 10:1) afforded product **4** as white solid (22 mg, 0.09 mmol, 44%; $^{19}$F NMR yield: 50%).

**$R_f$** (cyclohexane/EtOAc 8:2) = 0.21.

**M.p.** 38-39 °C.

**$^{1}$H NMR** (400 MHz, $CDCl_3$, 299 K) $\delta$ [ppm] = 8.00-7.92 (m, 2H, H3), 6.95-6.87 (m, 2H, H4), 4.82 (d, $^{2}J_{FH}$ = 47.6 Hz, 2H, H11), 4.32 (t, $^{3}J_{HH}$ = 6.2 Hz, 2H, H7), 3.86 (s, 3H, H1), 2.72 (td, $^{3}J_{HH}$ = 7.2 Hz, $^{4}J_{FH}$ = 2.5 Hz, 2H, H9), 2.11 (tt, $^{3}J_{HH}$ = 6.8, 6.5 Hz, 2H, H8).

**$^{13}$C NMR** (101 MHz, $CDCl_3$, 299 K) $\delta$ [ppm] = 206.3 (d, $^2J_{FC}$ = 19.8 Hz, 1C, C10), 166.4 (1C, C6), 163.6 (1C, C2), 131.7 (2C, C3), 122.6 (1C, C5), 113.8 (2C, C4), 85.1 (d, $^1J_{FC}$ = 184.9 Hz, 1C, C11), 63.7 (1C, C7), 55.6 (1C, C1), 34.9 (1C, C9), 22.17 (1C, C8).

**$^{19}$F NMR** (376 MHz, $CDCl_3$, 299 K) $\delta$ [ppm] = -227.7 (tt, $^2J_{FH}$ = 47.7 Hz, $^4J_{FH}$ = 2.5 Hz, 1F, F11).

**IR** (ATR) $\tilde{\upsilon}$ [cm$^{-1}$] = 2975 (w), 1737 (m), 1708 (s), 1602 (m), 1509 (m), 1466 (w), 1443 (w), 1420 (w), 1277 (s), 1256 (s), 1186 (w), 1165 (s), 1132 (m), 1118 (m), 1102 (s), 1084 (m), 1062 (m), 1017 (s), 1001 (s), 975 (m), 858 (s), 832 (w), 773 (s), 725 (w), 699 (m), 686 (m).

**HRMS** (ESI) *m/z* [M+Na]$^+$, calcd. for $C_{13}O_4FNa^+$: 277.0847, found: 277.0844.

**5-Fluoro-4-oxopentyl 4-nitrobenzoate (5)**

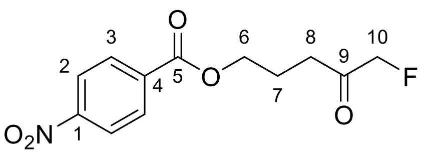


The reaction was performed according to **general procedure D** using pent-4-yn-1-yl 4-nitrobenzoate (**S3**) (47 mg, 0.2 mmol, 1.0 equiv). Column chromatography purification (cyclohexane/EtOAc 10:1) afforded product **5** as pale yellow solid (20 mg, 0.07 mmol, 38%; $^{19}$F NMR yield: 51%).

**$R_f$** (cyclohexane/EtOAc 8:2) = 0.18.

**M.p.** 69-70 °C.

**$^1$H NMR** (599 MHz, $CDCl_3$, 299 K) $\delta$ [ppm] = 8.31-8.27 (m, 2H, H2), 8.21-8.17 (m, 2H, H3), 4.82 (d, $^2J_{FH}$ = 47.7 Hz, 2H, H10), 4.42 (t, $^3J_{HH}$ = 6.3 Hz, 2H, H6), 2.76 (td, $^3J_{HH}$ = 7.1 Hz, $^4J_{FH}$ = 2.5 Hz, 2H, H8), 2.15 (tt, $^3J_{HH}$ = 7.1, 6.7 Hz, 2H, H7).

**$^{13}$C NMR** (151 MHz, $CDCl_3$, 299 K) $\delta$ [ppm] = 206.2 (d, $^3J_{FC}$ = 20.4 Hz, 1C, C9), 164.8 (1C, C5), 150.8 (1C, C1), 135.6 (1C, C4), 130.8 (2C, C3), 123.8 (2C, C2), 85.17 (d, $^1J_{FC}$ = 184.9 Hz, 1C, C10), 65.0 (1C, C6), 34.9 (1C, C8), 21.9 (1C, C7).

**$^{19}$F NMR** (564 MHz, $CDCl_3$, 299 K) $\delta$ [ppm] = -227.8 (tt, $^2J_{FH}$ = 47.6 Hz, $^4J_{FH}$ = 2.5 Hz, 1F, F10).

**IR** (ATR) $\tilde{\upsilon}$ [cm$^{-1}$] = 3117 (w), 1770 (w), 1709 (s), 1606 (w), 1518 (m), 1463 (w), 1432 (w), 1411 (w), 1354 (m), 1286 (s), 1161 (m), 1130 (m), 1052 (m), 1037 (s), 1015 (m), 970 (m), 878 (m), 840 (m), 786 (m), 718 (s).

**HRMS** (ESI) *m/z* [M+Na]$^+$, calcd. for $C_{12}H_{12}NO_5FNa^+$: 292.0592, found: 292.0593.

**5-Fluoro-4-oxohexyl benzoate (6)**

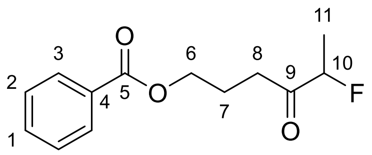


The reaction was performed according to **general procedure D** using hex-4-yn-1-yl benzoate (**S5**) (40 mg, 0.2 mmol, 1.0 equiv). Column chromatography purification (cyclohexane/EtOAc 10:1) afforded product **6** as colourless oil (22 mg, 0.09 mmol, 46%; $^{19}$F NMR yield: 51%).

**R$_f$** (cyclohexane/EtOAc 8:2) = 0.42.

**$^1$H NMR** (500 MHz, $CDCl_3$, 299 K) $\delta$ [ppm] = 8.05-8.01 (m, 2H, H3), 7.59-7.53 (m, 1H, H1), 7.47-7.41 (m, 2H, H2), 4.89 (dq, $^2J_{FH}$ = 49.4 Hz, $^3J_{HH}$ = 6.9 Hz, 1H, H10), 4.35 (t, $^3J_{HH}$ = 6.3 Hz, 2H, H6), 2.80 (td, $^3J_{HH}$ = 7.1 Hz, $^4J_{HF}$ = 2.8 Hz, 2H, H8), 2.09 (tt $^3J_{HH}$ = 6.8, 6.8 Hz, 2H, H7), 1.48 (dd, $^3J_{FH}$ = 24.0 Hz, $^3J_{HH}$ = 6.9 Hz, 3H).

**$^{13}$C NMR** (126 MHz, $CDCl_3$, 299 K) $\delta$ [ppm] = 209.4 (d, $^2J_{FC}$ = 24.4 Hz, 1C, C9), 166.6 (1C, C5), 133.12 (1C, C1), 130.3 (1C, C4), 129.7 (2C, C3), 128.5 (2C, C2) 92.7 (d, $^1J_{FC}$ = 181.0 Hz, 1C, C10), 64.1 (1C, C6), 34.0 (1C, C8), 22.1 (1C, C7), 17.7 (d, $^2J_{FC}$ = 22.0 Hz, 1C, C11).

**$^{19}$F NMR** (470 MHz, $CDCl_3$, 299 K) $\delta$ [ppm] = -184.5 (dqt, $^2J_{FH}$ = 49.5 Hz, $^3J_{FH}$ = 24.0 Hz, $^4J_{FH}$ = 2.8 Hz, 1F, F10).

**IR** (ATR) $\tilde{\upsilon}$ [cm$^{-1}$] = 2960 (w), 1716 (s), 1602 (w), 1584 (w), 1452 (w), 1372 (w), 1315 (w), 1271 (s), 1176 (w), 1115 (m), 1097 (m), 1070 (m), 1021 (m), 988 (w), 885 (w), 807 (w), 711 (s), 687 (w).

**HRMS** (ESI) *m/z* [M+Na]$^+$, calcd. for $C_{13}H_{15}O_3FNa^+$: 261.0897, found: 261.0894.

### 2-(5-Fluoro-4-oxopentyl)isoindoline-1,3-dione (7)

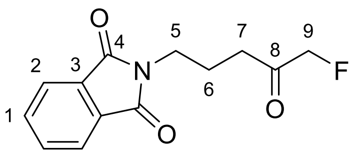


The reaction was performed according to **general procedure D** using 2-(pent-4-yn-1-yl)isoindoline-1,3-dione (**16**) (43 mg, 0.2 mmol, 1.0 equiv). Column chromatography purification (cyclohexane/EtOAc 8:1) afforded product **7** as white solid (32 mg, 0.13 mmol, 64%; $^{19}$F NMR yield: 72%).

**R$_f$** (cyclohexane/EtOAc 8:2) = 0.13.

**M.p.** 91-92 °C.

**$^1$H NMR** (500 MHz, $CDCl_3$, 299 K) $\delta$ [ppm] = 7.88-7.80 (m, 2H, H2), 7.76-7.68 (m, 2H, H1), 4.81 (d, $^2J_{FH}$ = 47.6 Hz, 2H, H9), 3.74 (t, $^3J_{HH}$ = 6.6 Hz, 2H, H5), 2.61 (td, $^3J_{HH}$ = 7.0 Hz, $^4J_{FH}$ = 2.3 Hz, 2H, H7), 2.02 (tt, $^3J_{HH}$ = 6.9, 6.8 Hz, 2H, H6).

**$^{13}$C NMR** (126 MHz, $CDCl_3$, 299 K) $\delta$ [ppm] = 206.0 (d, $^2J_{FC}$ = 19.8 Hz, 1C, C9), 168.6 (2C, C4), 134.2 (2C, C1), 132.2 (2C, C3), 123.4 (2C, C2), 85.1 (d, $^1J_{FC}$ = 184.7 Hz, 1C, C9), 37.1 (1C, C5), 35.4 (1C, C8), 21.8 (1C, C6).

**$^{19}$F NMR** (470 MHz, $CDCl_3$, 299 K) $\delta$ [ppm] = -228.0 (tt, $^2J_{FH}$ = 47.6 Hz, $^4J_{FH}$ = 2.4 Hz, 1F, F9).

**IR** (ATR) $\tilde{\upsilon}$ [cm$^{-1}$] = 2938 (w), 1770 (w), 1732 (m), 1702 (s), 1614 (w), 1462 (m), 1439 (m), 1401 (s), 1373 (m), 1363 (m), 1335 (m), 1296 (w), 1286 (w), 1265 (w), 1255 (w), 1105 (m), 1073 (m), 1044 (m), 999 (m), 971 (m), 886 (m), 860 (w), 794 (w), 715 (s), 722 (s), 686 (w).

**HRMS** (ESI) *m/z* [M+Na]$^+$, calcd. for $C_{13}H_{12}NO_3FNa^+$: 272.0693, found: 272.0694.

# V. SAR analysis of score scaffold

**General procedure E for SAR analysis of score scaffold**

The alkyne (0.2 mmol, 1.0 equiv) and *p*-TolI (8.7 mg, 40 µmol, 20 mol %) were dissolved in $CHCl_3$ (0.5 mL) in a Teflon® screw cap vial. $H_2O$ (3.6 µL, 0.2 mmol, 1.0 equiv), amine/HF 1:7.5 (0.5 mL), and Selectfluor® (106 mg, 0.3 mmol, 1.5 equiv) were added, and the reaction mixture was stirred for 24 h at ambient temperature. The reaction mixture was poured into a saturated solution of $NaHCO_3$ and the aqueous layer was extracted with $CH_2Cl_2$ (3×). The combined organic layers were dried over $MgSO_4$, filtered and the solvent was removed in vacuo. The yield was determined by $^{19}F$ NMR analysis of the crude reaction mixture using ethyl fluoroacetate as internal standard. The observed α-fluoroketones were assigned by analogy to previously reported α-fluoroketones in this paper.

**4-Fluoro-3-oxobutyl benzoate (8)**

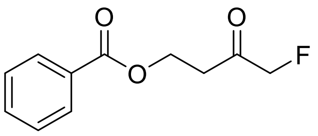


The reaction was performed according to **general procedure E** using but-3-yn-1-yl benzoate (**S6**, 35 mg, 0.2 mmol, 1.0 equiv). A $^{19}F$ NMR yield of 16% was observed.

**6-Fluoro-5-oxohexyl benzoate (9)**

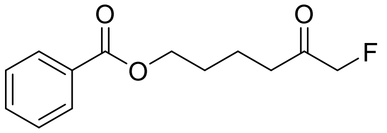


The reaction was performed according to **general procedure E** using but-3-yn-1-yl benzoate (**14**, 40 mg, 0.2 mmol, 1.0 equiv). A $^{19}F$ NMR yield less than 5% was observed.

**5-(Benzyloxy)-1-fluoropentan-2-one (10)**

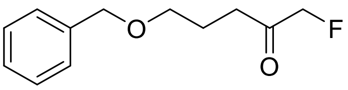

The reaction was performed according to **general procedure E** using (pent-4-yn-1-yloxy)methyl)benzene (**S7**, 35 mg, 0.2 mmol, 1.0 equiv). A $^{19}F$ NMR yield less than 5% was observed.

**5-Fluoro-4-oxopentyl acetate (11)**

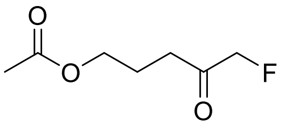

The reaction was performed according to **general procedure E** using pent-4-yn-1-yl acetate (**S8**, 25 mg, 0.2 mmol, 1.0 equiv). A $^{19}F$ NMR yield of 16% was observed.

***N*-(5-Fluoro-4-oxopentyl)benzamide (12)**

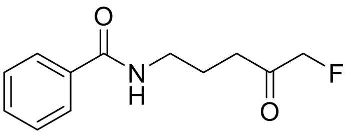

The reaction was performed according to **general procedure E** using *N*-(pent-4-yn-1-yl)benzamide (**S9**, 37 mg, 0.2 mmol, 1.0 equiv). A $^{19}F$ NMR yield of 15% was observed.

***N*-(5-Fluoro-4-oxopentyl)-*N*-methylbenzamide (13)**

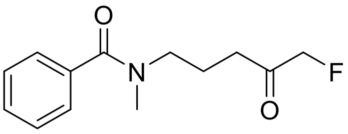

The reaction was performed according to **general procedure E** using *N*-methyl-*N*-(pent-4-yn-1-yl)benzamide (**S10**, 40 mg, 0.2 mmol, 1.0 equiv) as starting material. A $^{19}F$ NMR yield of 15% was observed.

# VI. Mechanistic and kinetic studies

### Addition of methyl benzoate to the reaction

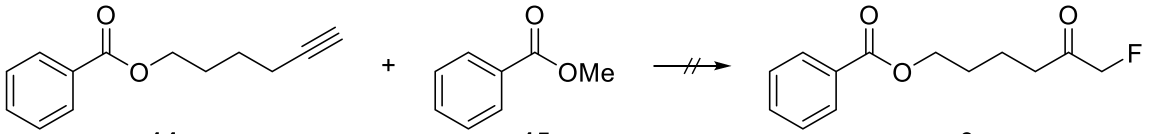


**14** **15** **9**

<5%

The reaction was performed according to **general procedure E** using hex-5-yn-1-yl benzoate (**14**, 40 mg, 0.2 mmol, 1.0 equiv) and methyl benzoate (**15**, 25 µL, 0.2 mmol, 1.0 equiv) as additive. A $^{19}F$ NMR yield less than 5% was observed.

### Mass experiment with labelled $H_2{}^{18}O$

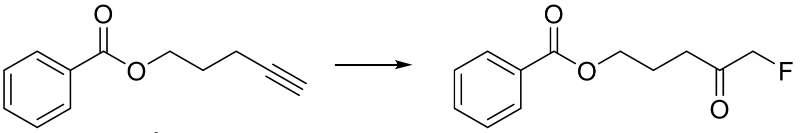


**1** **$^{16}$2 : $^{18}$2**

85 : 15

The reaction was performed according to **general procedure E** using pent-4-yn-1-yl benzoate (**1**, 40 mg, 0.2 mmol, 1.0 equiv) and $H_2{}^{18}O$ (3.6 µL, 0.2 mmol, 1.0 equiv). Column chromatography purification (cyclohexane/EtOAc 10:1) afforded product **2** as colourless oil (25 mg, 0.11 mmol, 56%; $^{19}F$ NMR yield: 64%). A ESI$^{+}$–HRMS experiment showed a ratio of 85:15 towards the product **$^{16}$2** and **$^{18}$2**, indicating that the added water played a role in the reaction (natural abundance of $^{18}O$ is <1%) [17].

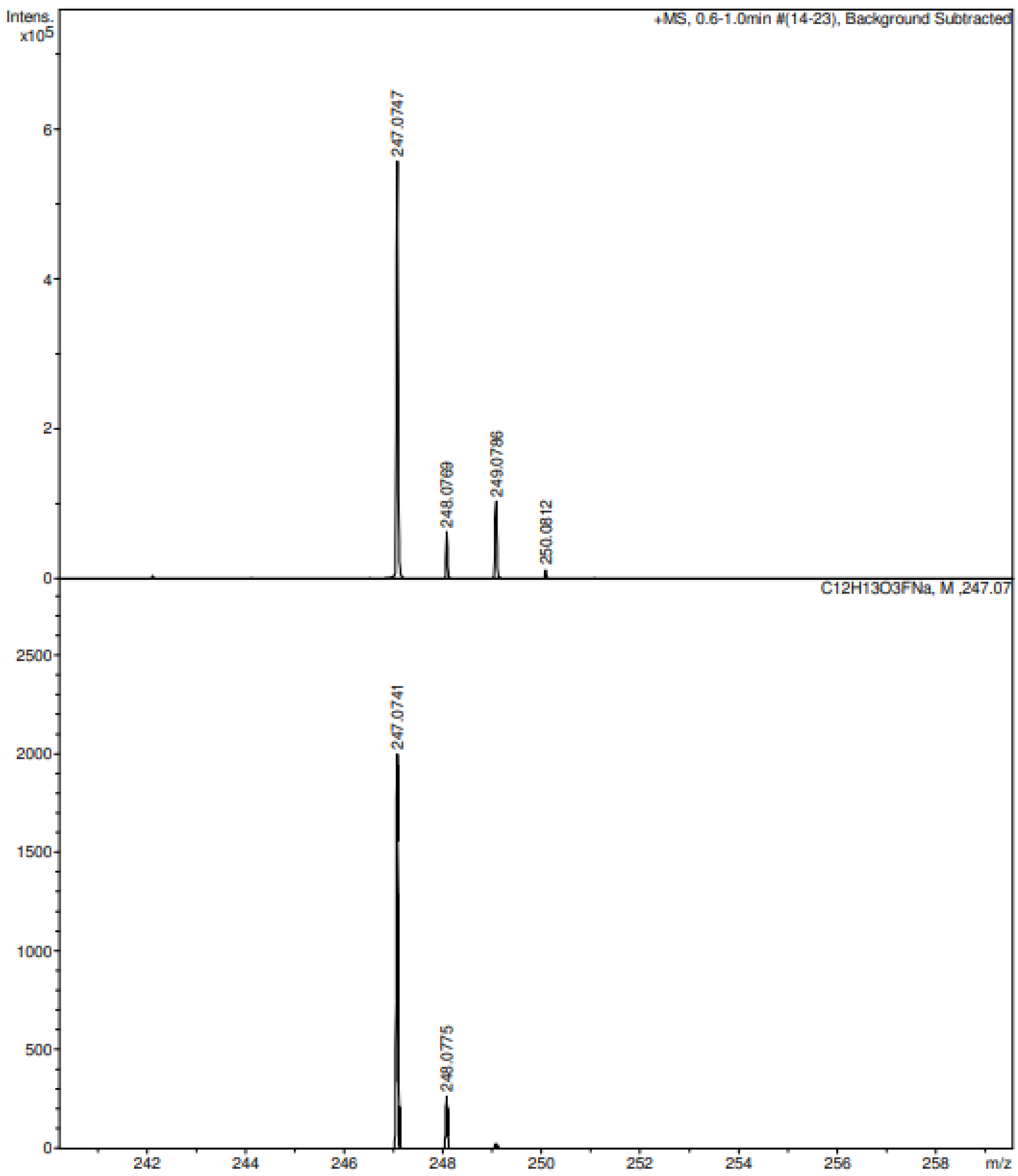


**Figure S1:** Mass experiment with labelled $H_2{}^{18}O$.

# LabelChecker Results

Formula: C12 H13 O3 F Na
Mass (monoisotopic): 247.07

Difference Value: 0.000365
Error Sum: 0.019
Error (%): 0.558

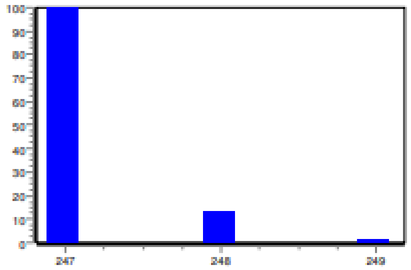


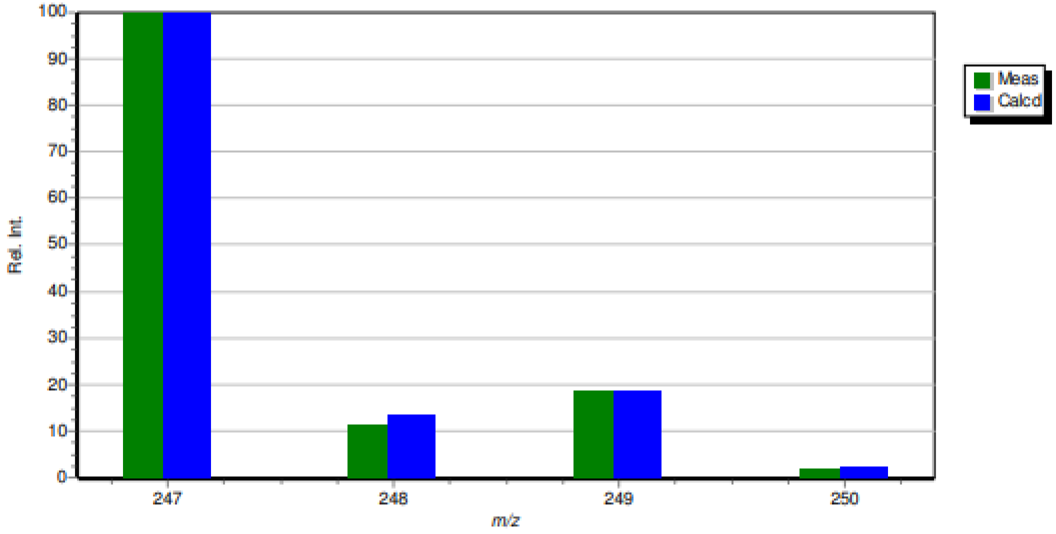


Oxygen-18: 0-fold (%): 100.00 85.41
Oxygen-18: 1-fold (%): 17.09 14.59
Oxygen-18: 2-fold (%): 0.00 0.00
Label Atom Sum: 0.15 (4.86%)

Isotope List used for fitting data:

| m/z | intensity |
|---|---|
| 247.0747 | 557115 |
| 248.0769 | 63204 |
| 249.0786 | 103508 |
| 250.0812 | 12036 |
| 251.0713 | 1600 |

**Figure S2:** Mass experiment with labelled $H_2{}^{18}O$; LabelChecker results.

In addition, the obtained product was submitted to $^{13}$C NMR analysis (151 MHz, $CDCl_3$) to investigate, if the aryl ester or the ketone contains the added $H_2{}^{18}O$. From the mass experiment it was assumed that the obtained product would show an isotopic shift of one of the carbonyls groups with a ratio of 85:15 for **$^{16}$2** and **$^{18}$2**. The corresponding signal for the ketone ($\delta_{CO}$ = 206.15 ppm) did not show an additional peak nearby. However, the carbonyl for the aryl ester showed another peak close to the initial signal ($\delta_{CO}$ = 166.50 ppm vs. $\delta_{C18O}$ =166.47 ppm), with a shift of 4.42 Hz [18]. Integration of both peaks revealed a ratio of 85:15 which is in accordance with the mass experiment.

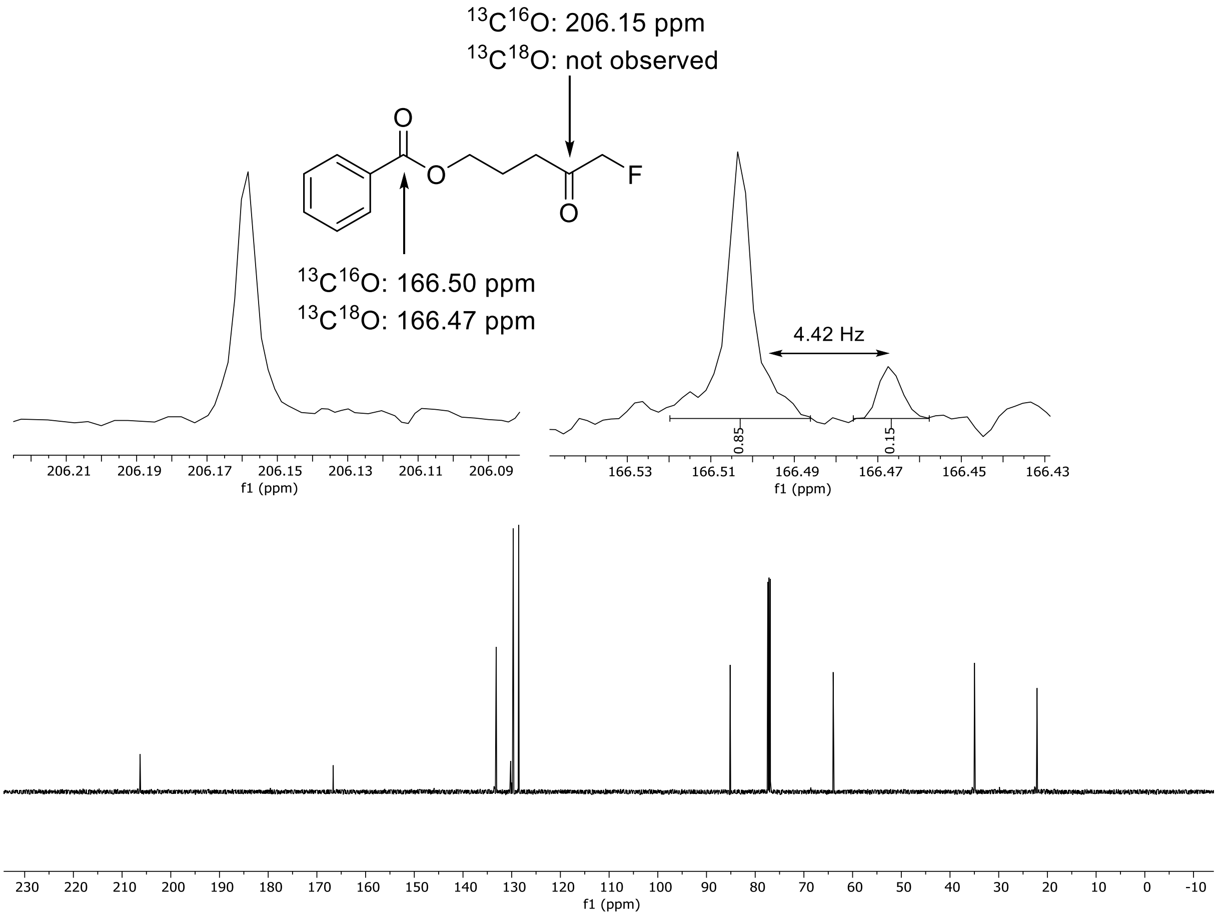

**Stoichiometric reaction with freshly prepared difluoro(*p*-tolyl)-$\lambda^3$-iodane**

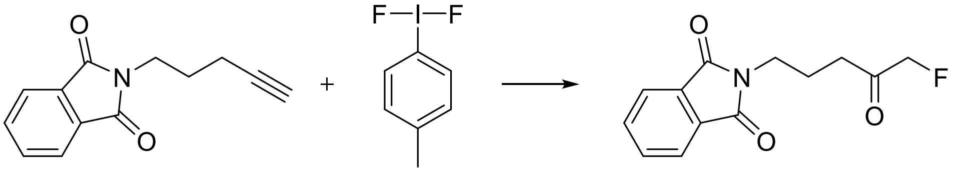

**16** **17** **7**

without amine:HF <5%

with amine:HF 1:7.5 76%

Experiment without amine/HF:

2-(Pent-4-yn-1-yl)isoindoline-1,3-dione (**16**, 22 mg, 0.1 mmol, 1.0 equiv) and difluoro(*p*-tolyl)-$\lambda^3$-iodane (**17**, 26 mg, 0.1 mmol, 1.0 equiv) were dissolved in $CHCl_3$ (0.5 mL) in a Teflon® screw cap vial, and the reaction mixture was stirred for 24 h at ambient temperature. The reaction mixture was poured into a saturated solution of $NaHCO_3$ and the aqueous layer was extracted with $CH_2Cl_2$ (3×). The combined organic layers were dried over $MgSO_4$, filtered, and the solvent was removed in vacuo. A $^{19}F$ NMR yield of <5% was observed (determined by $^{19}F$ NMR analysis of the crude reaction mixture using ethyl fluoroacetate as internal standard).

Experiment with amine/HF 1:7.5:

2-(Pent-4-yn-1-yl)isoindoline-1,3-dione (**16**, 22 mg, 0.1 mmol, 1.0 equiv) and difluoro(*p*-tolyl)-$\lambda^3$-iodane (**17**, 26 mg, 0.1 mmol, 1.0 equiv) were dissolved in $CHCl_3$ (0.25 mL), and amine/HF 1:7.5 (0.25 mL) in a Teflon® screw cap vial. The reaction mixture was stirred for 24 h at ambient temperature. The reaction mixture was poured into a saturated solution of $NaHCO_3$ and the aqueous layer was extracted with $CH_2Cl_2$ (3×). The combined organic layers were dried over $MgSO_4$, filtered, and the solvent was removed in vacuo. A $^{19}F$ NMR yield of 76% was observed (determined by $^{19}F$ NMR analysis of the crude reaction mixture using ethyl fluoroacetate as internal standard).

## Hammett correlation for the substrate

The corresponding alkyne (0.2 mmol, 1.0 equiv) and *p*-TolI (8.7 mg, 40 µmol, 20 mol %) were dissolved in $CHCl_3$ (0.5 mL) in a Teflon® screw cap vial. $H_2O$ (3.6 µL, 0.2 mmol, 1.0 equiv), amine/HF 1:7.5 (0.5 mL), and Selectfluor® (106 mg, 0.3 mmol, 1.5 equiv) were added, and the reaction mixture was stirred for 15, 30, 45, or 60 min at ambient temperature. The reaction mixture was poured into a saturated solution of $NaHCO_3$ and the aqueous layer was extracted with $CH_2Cl_2$ (3×). The combined organic layers were dried over $MgSO_4$, filtered, and the solvent was removed in vacuo. The yield was determined by $^{19}F$ NMR analysis of the crude reaction mixture using ethyl fluoroacetate as internal standard.

**Table S3:** Kinetic studies on pent-4-yn-1-yl benzoate **(1)**.

| time | yield | linear correlation |
|---|---|---|
| 0 min | 0% | |
| 15 min | 2% | |
| 30 min | 11% | |
| 45 min | 14% | |
| 60 min | 20% | |

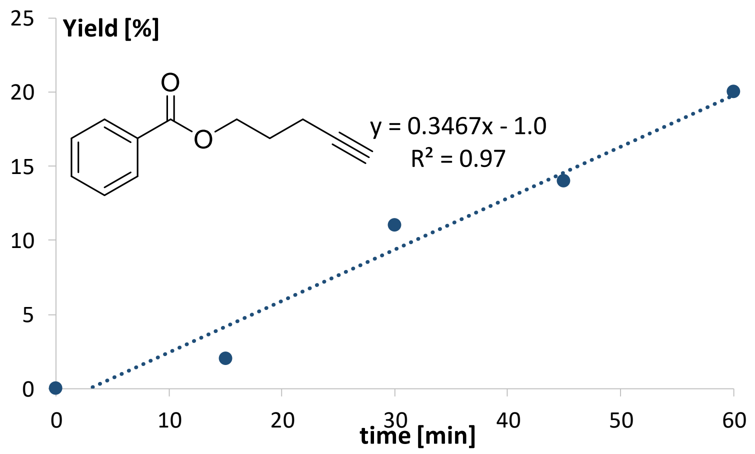

**Table S4:** Kinetic studies on pent-4-yn-1-yl 4-chlorobenzoate **(S1)**.

| time | yield | linear correlation |
|---|---|---|
| 0 min | 0% | 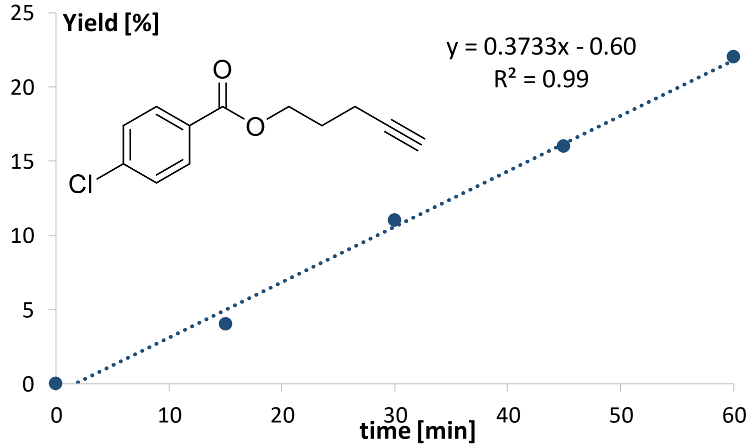 |
| 15 min | 4% | |
| 30 min | 11% | |
| 45 min | 16% | |
| 60 min | 21% | |



**Table S5:** Kinetic studies on pent-4-yn-1-yl 4-methoxybenzoate **(S2)**.

| time | yield | linear correlation |
|---|---|---|
| 0 min | 0% | 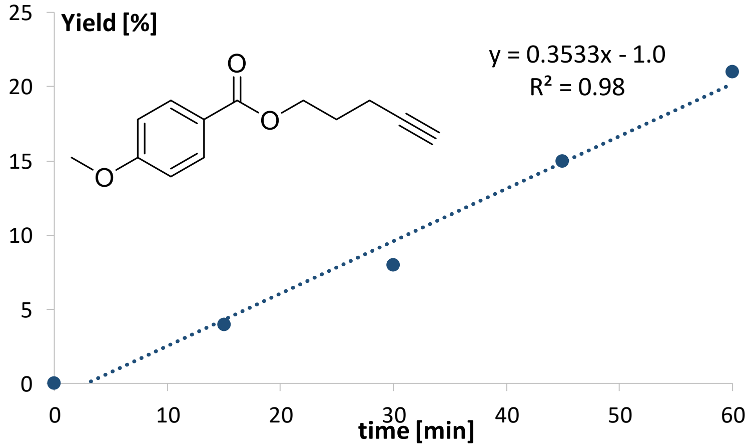 |
| 15 min | 4% | |
| 30 min | 8% | |
| 45 min | 14% | |
| 60 min | 21% | |

**Table S6:** Kinetic studies on pent-4-yn-1-yl 4-nitrobenzoate **(S3)**.

| time | yield | linear correlation |
|---|---|---|
| 0 min | 0% | 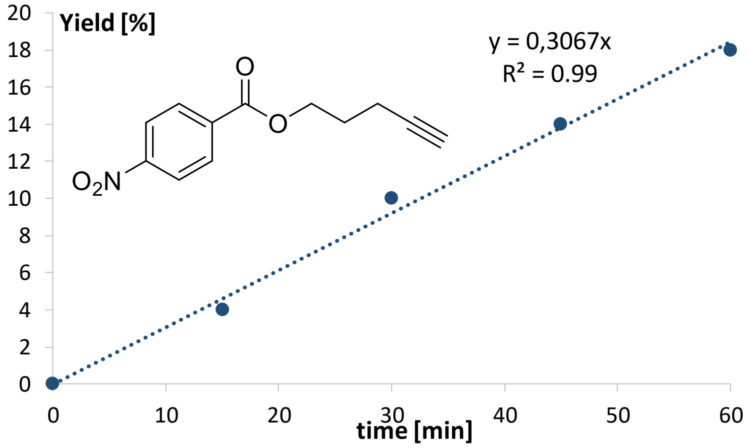 |
| 15 min | 4% | |
| 30 min | 10% | |
| 45 min | 14% | |
| 60 min | 18% | |



**Table S7:** Hammett plot of the substrate.

| R | $\sigma_p$ | linear correlation |
|---|---|---|
| OMe | -0.26 | 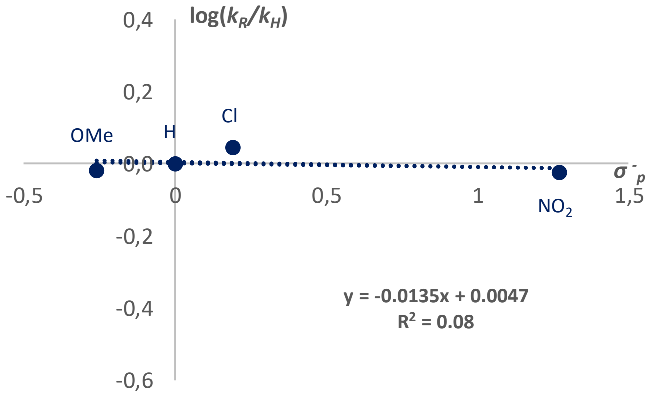 |
| H | 0.00 | |
| Cl | 0.19 | |
| $NO_2$ | 1.27 | |

## Hammett correlation for the catalyst

Pent-4-yn-1-yl benzoate (**1**) (38 mg, 0.2 mmol, 1.0 equiv) and the corresponding catalyst (40 µmol, 20 mol %) were dissolved in $CHCl_3$ (0.5 mL) in a Teflon® screw cap vial. $H_2O$ (3.6 µL, 0.2 mmol, 1.0 equiv), amine/HF 1:7.5 (0.5 mL), and Selectfluor® (106 mg, 0.3 mmol, 1.5 equiv) were added, and the reaction mixture was stirred for 15, 30, 45, or 60 min at ambient temperature. The reaction mixture was poured into a saturated solution of $NaHCO_3$ and the aqueous layer was extracted with $CH_2Cl_2$ (3×). The combined organic layers were dried over $MgSO_4$, filtered, and the solvent was removed in vacuo. The yield was determined by $^{19}F$ NMR analysis of the crude reaction mixture using ethy fluoroacetate as internal standard.

**Table S8:** Kinetic studies on *p*-iodotoluene.

| time | yield | linear correlation |
|---|---|---|
| 0 min | 0% | 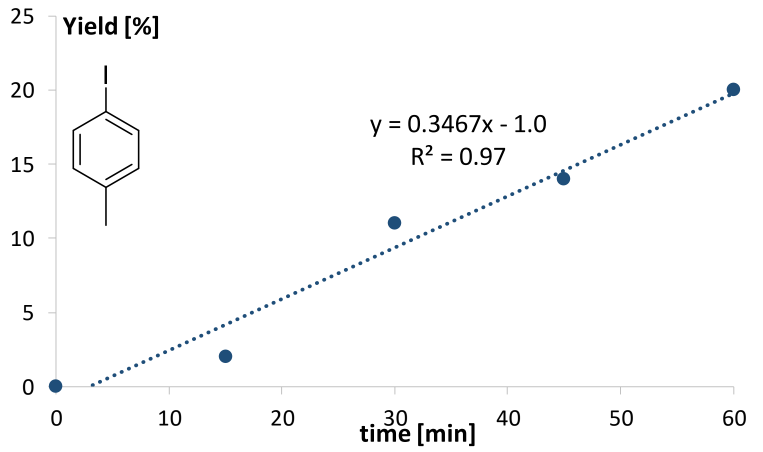 |
| 15 min | 2% | |
| 30 min | 11% | |
| 45 min | 14% | |
| 60 min | 20% | |

**Table S9:** Kinetic studies on iodobenzene.

| time | yield | linear correlation |
|---|---|---|
| 0 min | 0% | 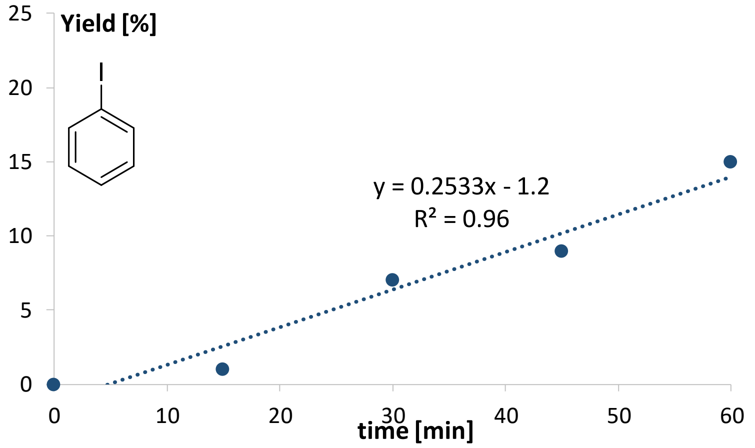 |
| 15 min | 1% | |
| 30 min | 7% | |
| 45 min | 9% | |
| 60 min | 15% | |

**Table S10:** Kinetic studies on *p*-chloroiodobenzene.

| time | yield | linear correlation |
|---|---|---|
| 0 min | 0% | 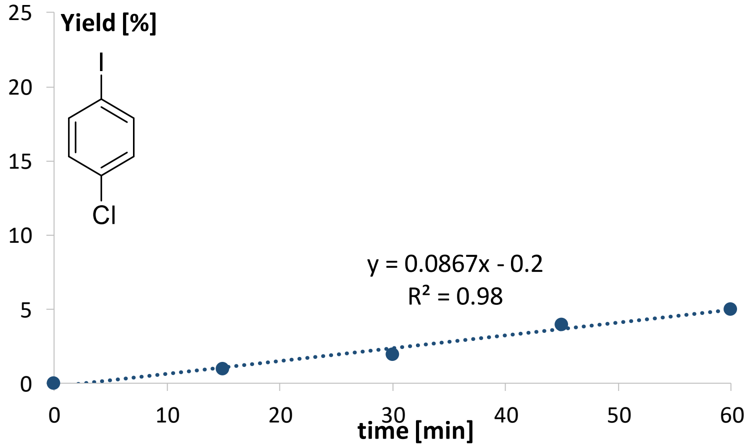 |
| 15 min | 1% | |
| 30 min | 2% | |
| 45 min | 4% | |
| 60 min | 5% | |

**Table S11:** Kinetic studies on *p*-bromoiodobenzene.

| time | yield | linear correlation |
|---|---|---|
| 0 min | 0% | 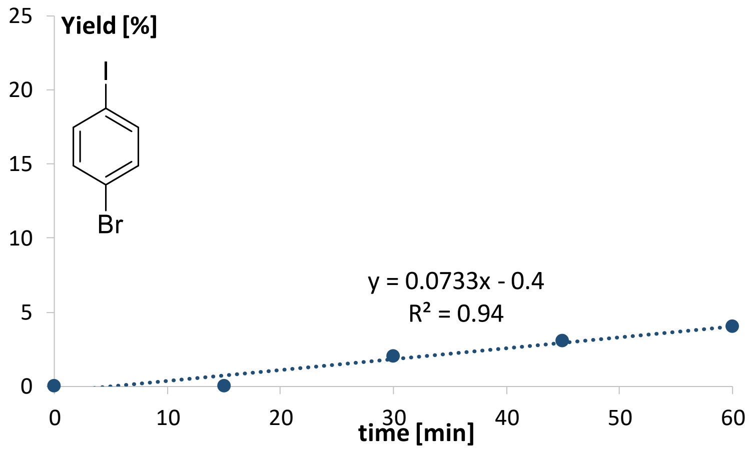 |
| 15 min | 0% | |
| 30 min | 2% | |
| 45 min | 3% | |
| 60 min | 4% | |



**Table S12:** Hammett plot of the catalyst.

| R | $\sigma^{-}_{p}$ | linear correlation |
|---|---|---|
| Me | -0.17 | 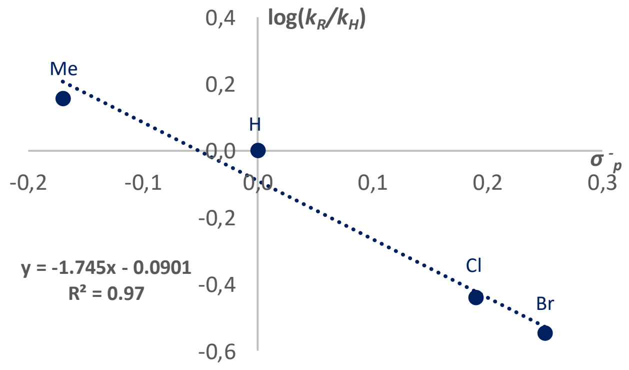 |
| H | 0.00 | |
| Cl | 0.19 | |
| Br | 0.25 | |

# VII. X-ray crystallographic data

### X-ray diffraction

Data sets for compound **2** were collected with a Bruker D8 Venture CMOS diffractometer. Programs used: data collection: APEX3 V2016.1-0 (Bruker AXS Inc., **2016**); cell refinement: SAINT V8.37A (Bruker AXS Inc., **2015**); data reduction: SAINT V8.37A (Bruker AXS Inc., **2015**); absorption correction, SADABS V2014/7 (Bruker AXS Inc., **2014**); structure solution *SHELXT-2015* (Sheldrick, G. M. *Acta Cryst.,* **2015**, *A71*, 3-8); structure refinement *SHELXL-2015* (Sheldrick, G. M. *Acta Cryst.,* **2015**, *C71* (1), 3-8) and graphics, *XP* (Version 5.1, Bruker AXS Inc., Madison, Wisconsin, USA, **1998**). *R*-values are given for observed reflections, and $wR^2$ values are given for all reflections.

### X-ray crystal structure analysis of 2 (gil9678)

A colourless plate-like specimen of $C_{12}H_{13}FO_3$, approximate dimensions 0.039 mm × 0.138 mm × 0.190 mm, was used for the X-ray crystallographic analysis. The X-ray intensity data were measured. A total of 1105 frames were collected. The total exposure time was 18.11 hours. The frames were integrated with the Bruker SAINT software package using a wide-frame algorithm. The integration of the data using a monoclinic unit cell yielded a total of 15346 reflections to a maximum θ angle of 66.51° (0.84 Å resolution), of which 1896 were independent (average redundancy 8.094, completeness = 99.7%, $R_{int}$ = 6.64%, $R_{sig}$ = 4.35%) and 1494 (78.80%) were greater than $2\sigma(F^2)$. The final cell constants of $a$ = 11.6230(3) Å, $b$ = 5.63180(10) Å, $c$ = 16.4197(4) Å, β = 91.309(2)°, volume = 1074.53(4) Å$^3$, are based upon the refinement of the XYZ-centroids of 6035 reflections above 20 σ(I) with 10.77° < 2θ < 132.4°. Data were corrected for absorption effects using the multi-scan method (SADABS). The ratio of minimum to maximum apparent transmission was 0.752. The calculated minimum

and maximum transmission coefficients (based on crystal size) are 0.8430 and 0.9650. The structure was solved and refined using the Bruker SHELXTL Software Package, using the space group *P*12$_1$/*c*1, with *Z* = 4 for the formula unit, $C_{12}H_{13}FO_3$. The final anisotropic full-matrix least-squares refinement on $F^2$ with 145 variables converged at R1 = 4.61%, for the observed data and wR2 = 11.55% for all data. The goodness-of-fit was 1.060. The largest peak in the final difference electron density synthesis was 0.176 $e^-/Å^3$ and the largest hole was −0.194 $e^-/Å^3$ with an RMS deviation of 0.042 $e^-/Å^3$. On the basis of the final model, the calculated density was 1.386 $g/cm^3$ and F(000), 472 $e^-$. CCDC number: 2000136.

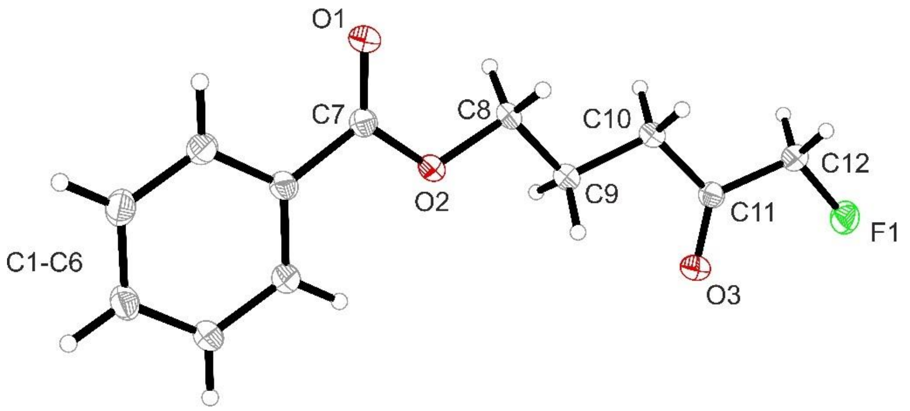


**Figure S3:** Crystal structure of compound **2**. Thermal ellipsoids are shown at 30% probability.


1. *APEX3* (**2016**), *SAINT* (**2015**) and *SADABS* (**2015**), Bruker AXS Inc., Madison, Wisconsin, USA.
2. Sheldrick, G. M., *SHELXT – Integrated space-group and crystal-structure determination*, *Acta Cryst.,* **2015**, *A71*, 3-8.
3. Sheldrick, G.M., *Crystal structure refinement with SHELXL*, *Acta Cryst.*, **2015**, *C71 (1)*, 3-8.
4. *XP – Interactive molecular graphics, Version 5.1,* Bruker AXS Inc., Madison, Wisconsin, USA, **1998**.

# VIII. NMR spectra of unreported starting materials

**_N_-Methyl-_N_-(pent-4-yn-1-yl)benzamide (S10)**

**$^{1}$H NMR** (500 MHz, DMSO-$d_6$, 373 K)

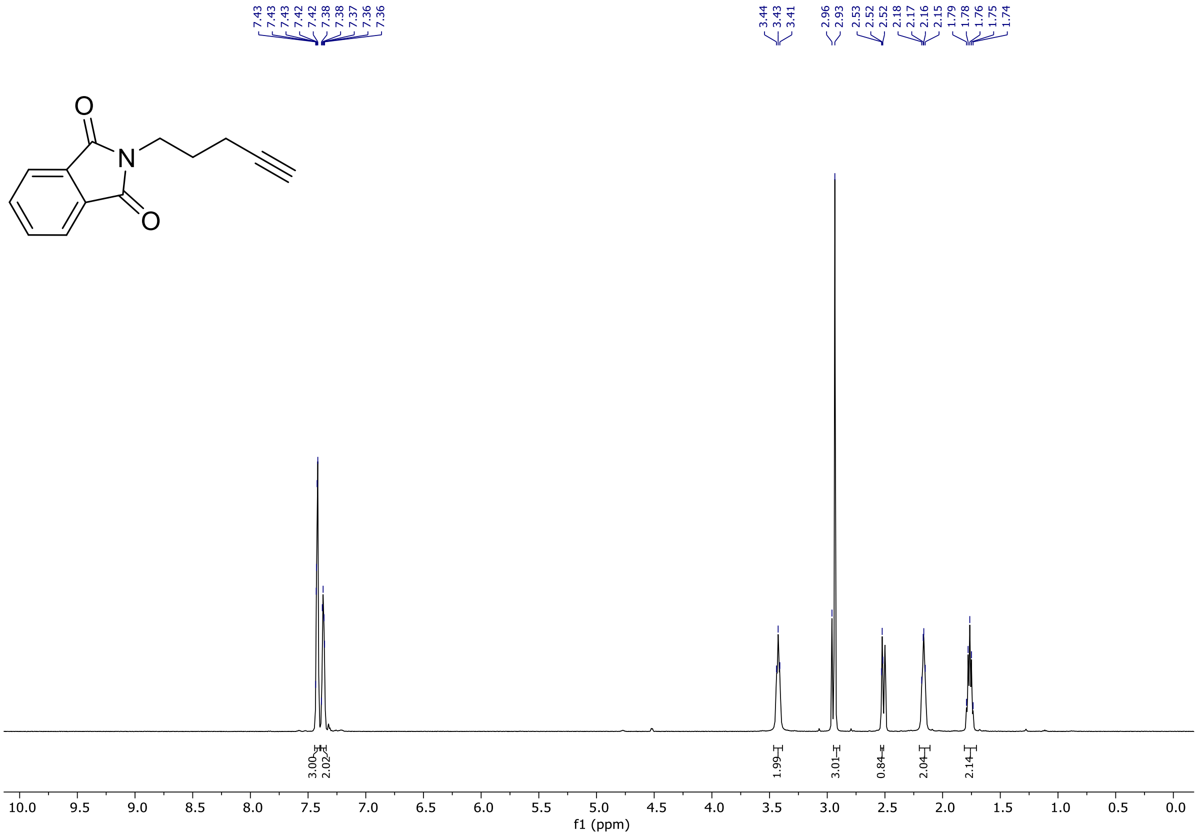


**$^{13}$C NMR (**126 MHz, DMSO-$d_6$, 373 K)

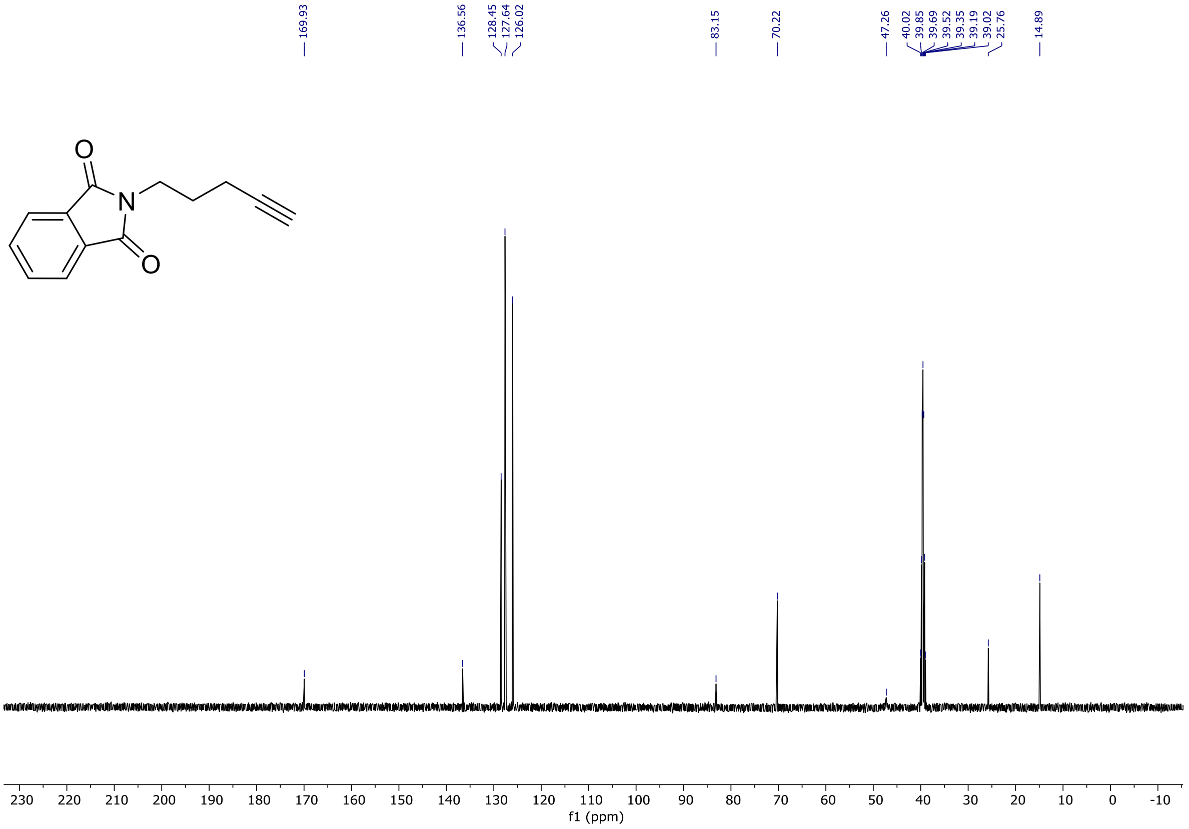

# IX. NMR spectra of α-fluoroketones

## 5-Fluoro-4-oxopentyl benzoate (2)

**$^{1}$H NMR** (500 MHz, $CDCl_3$, 299 K)

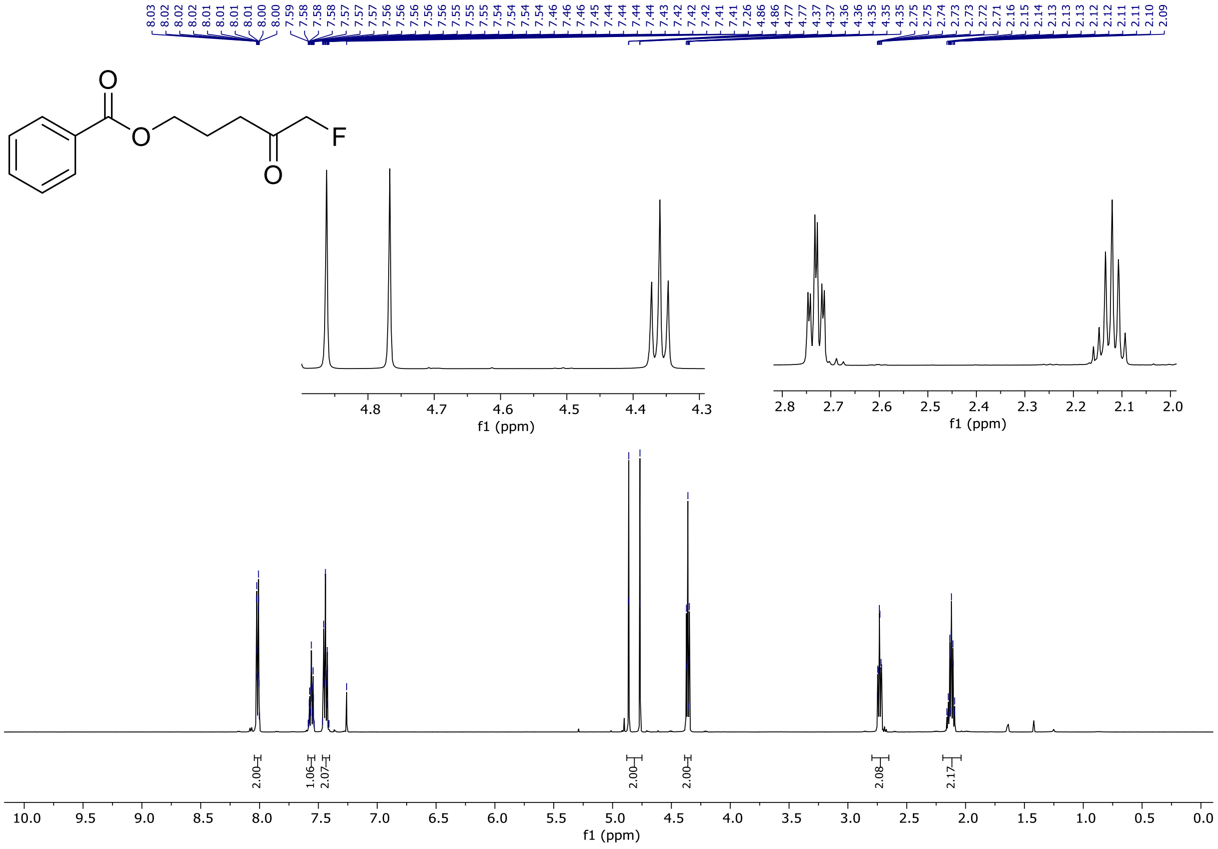

**$^{13}$C NMR** (126 MHz, $CDCl_3$, 299 K)

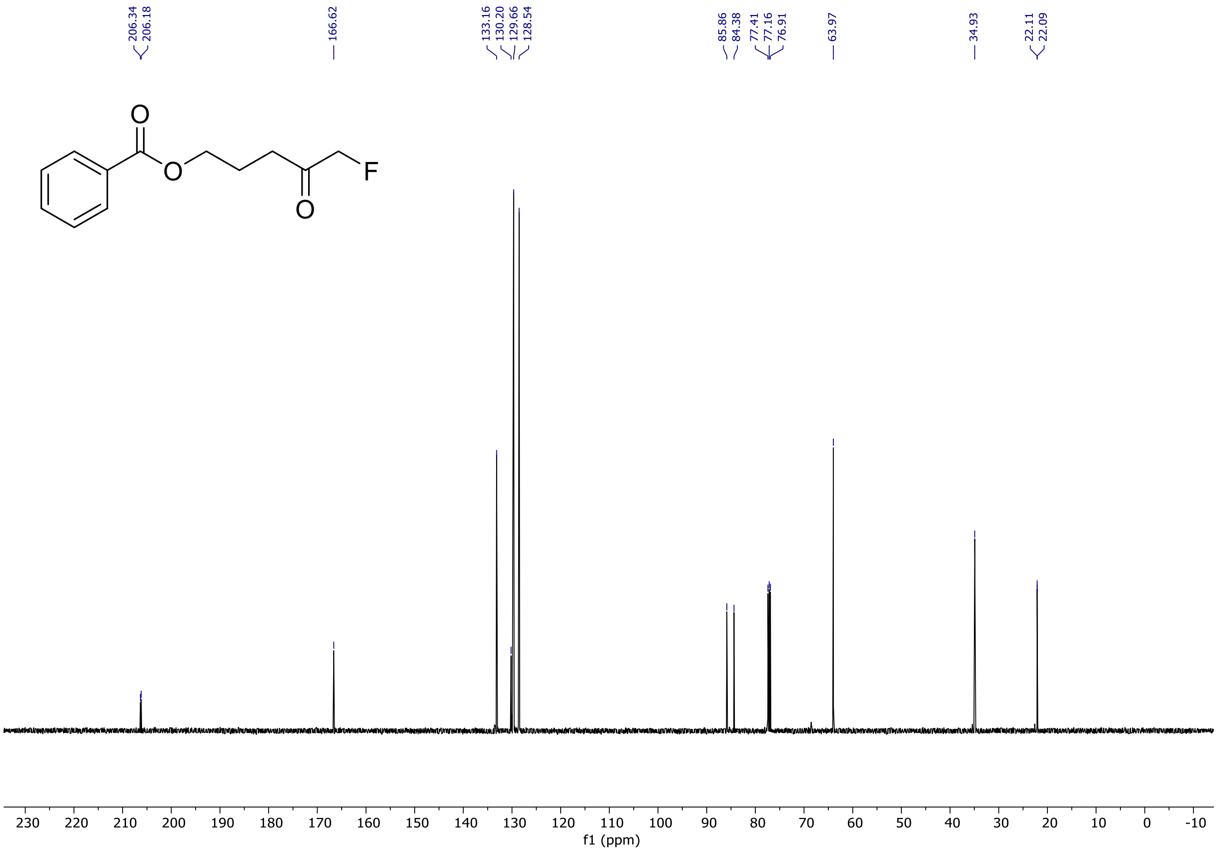


**$^{19}$F NMR** (470 MHz, $CDCl_3$, 299 K)

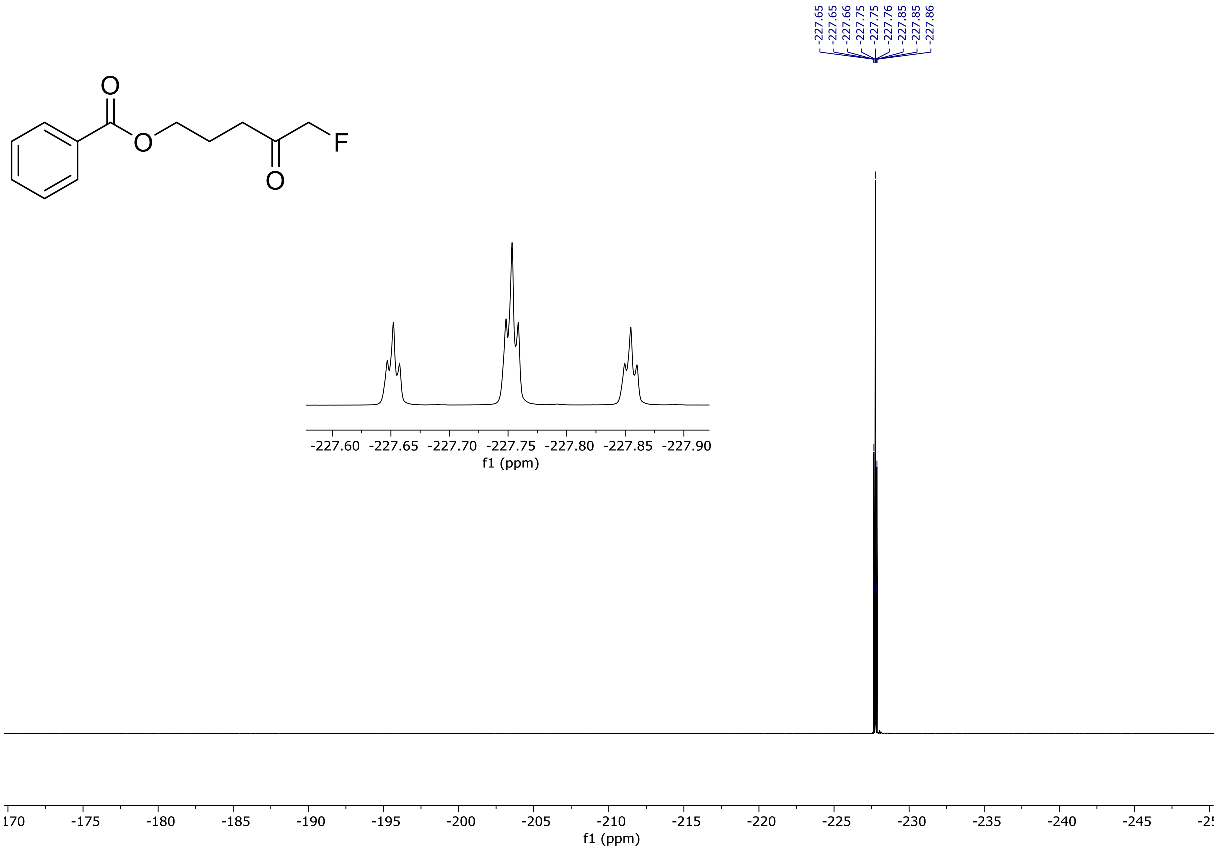

## 5-Fluoro-4-oxopentyl 4-chlorobenzoate (3)

**$^1$H NMR** (599 MHz, $CDCl_3$, 299 K)

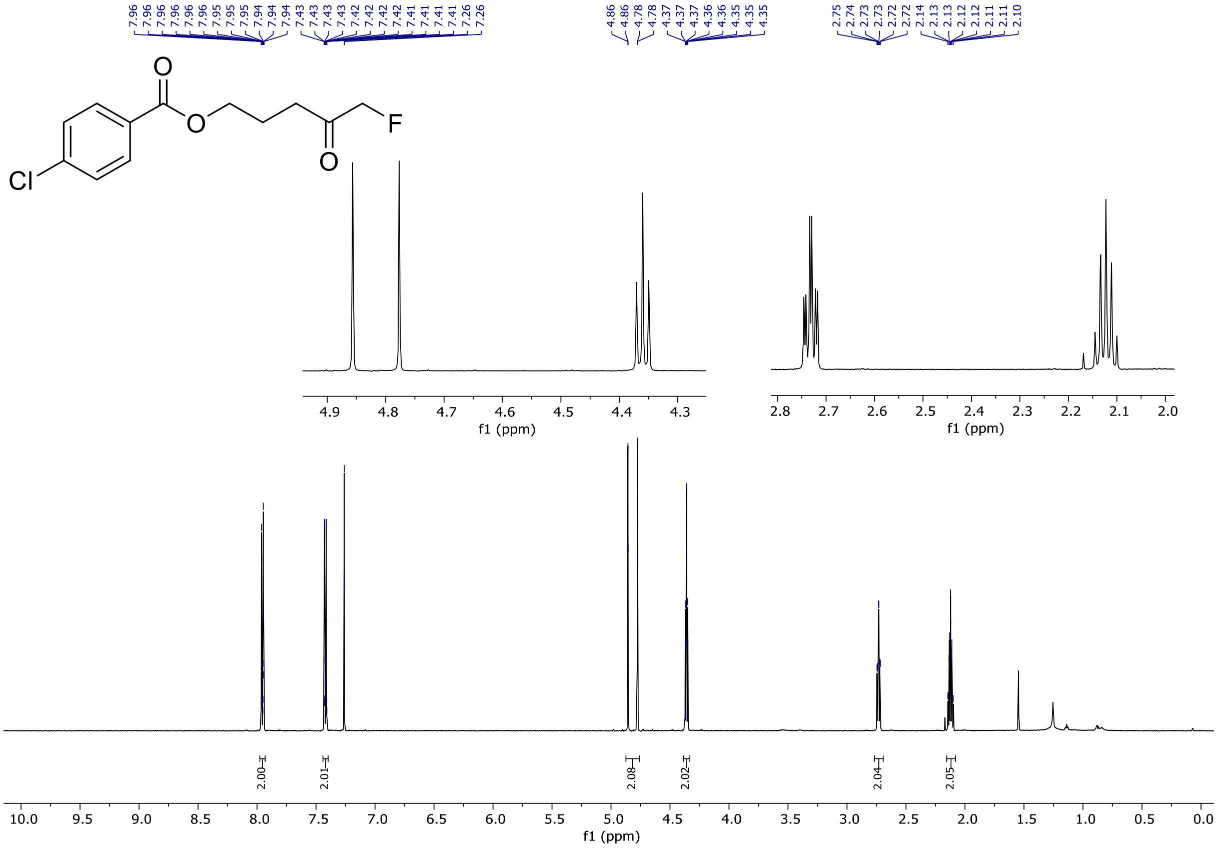


**$^{13}$C NMR** (151 MHz, $CDCl_3$, 299 K)

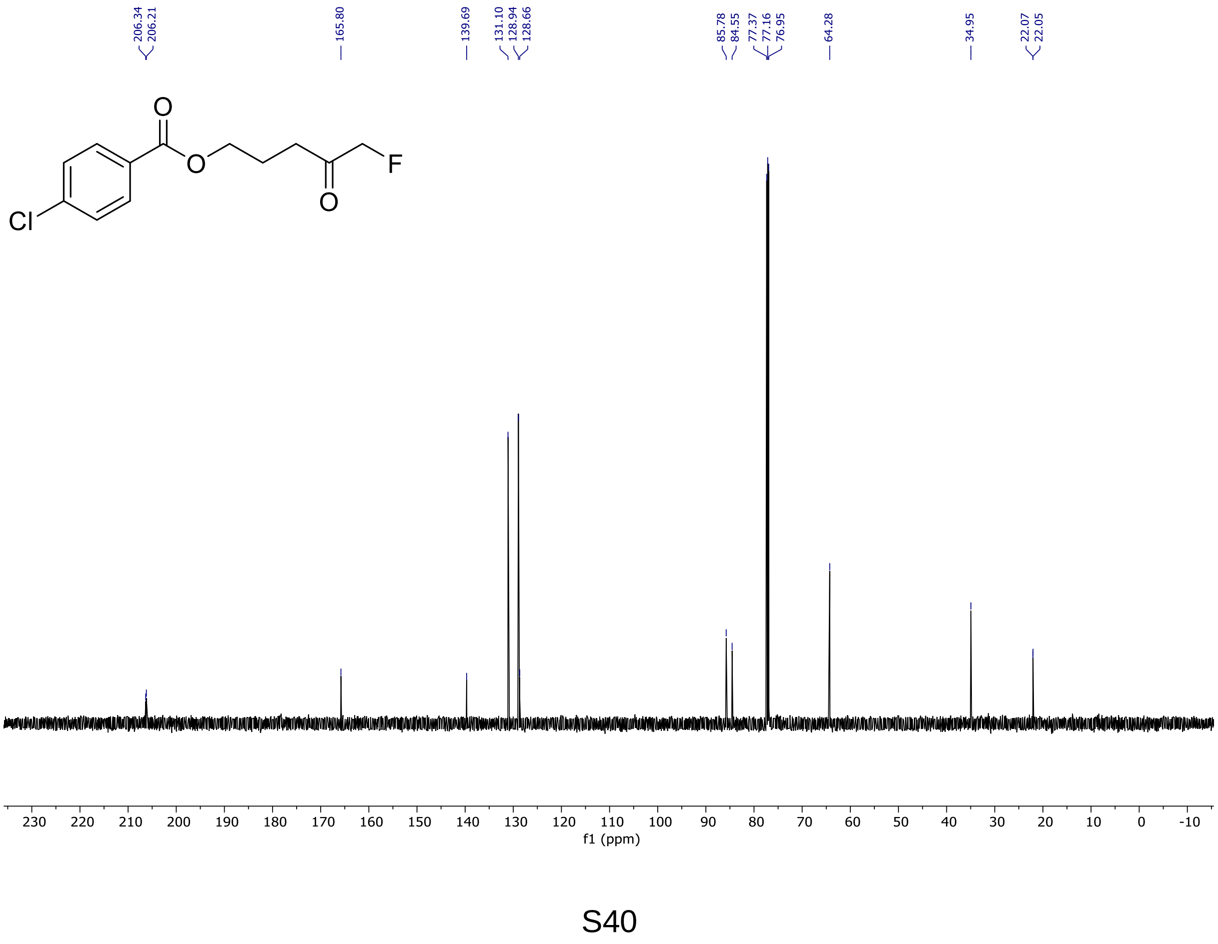

**$^{19}$F NMR** (564 MHz, $CDCl_3$, 299 K)

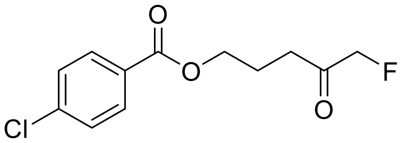


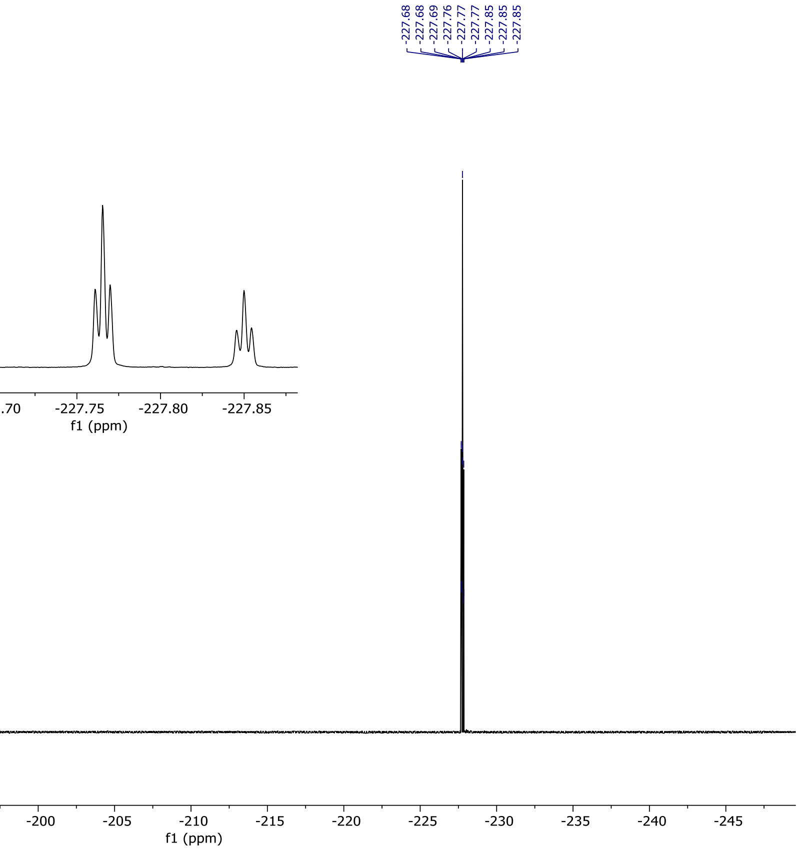

## 5-Fluoro-4-oxopentyl 4-methoxybenzoate (4)

**$^{1}$H NMR** (400 MHz, $CDCl_3$, 299 K)

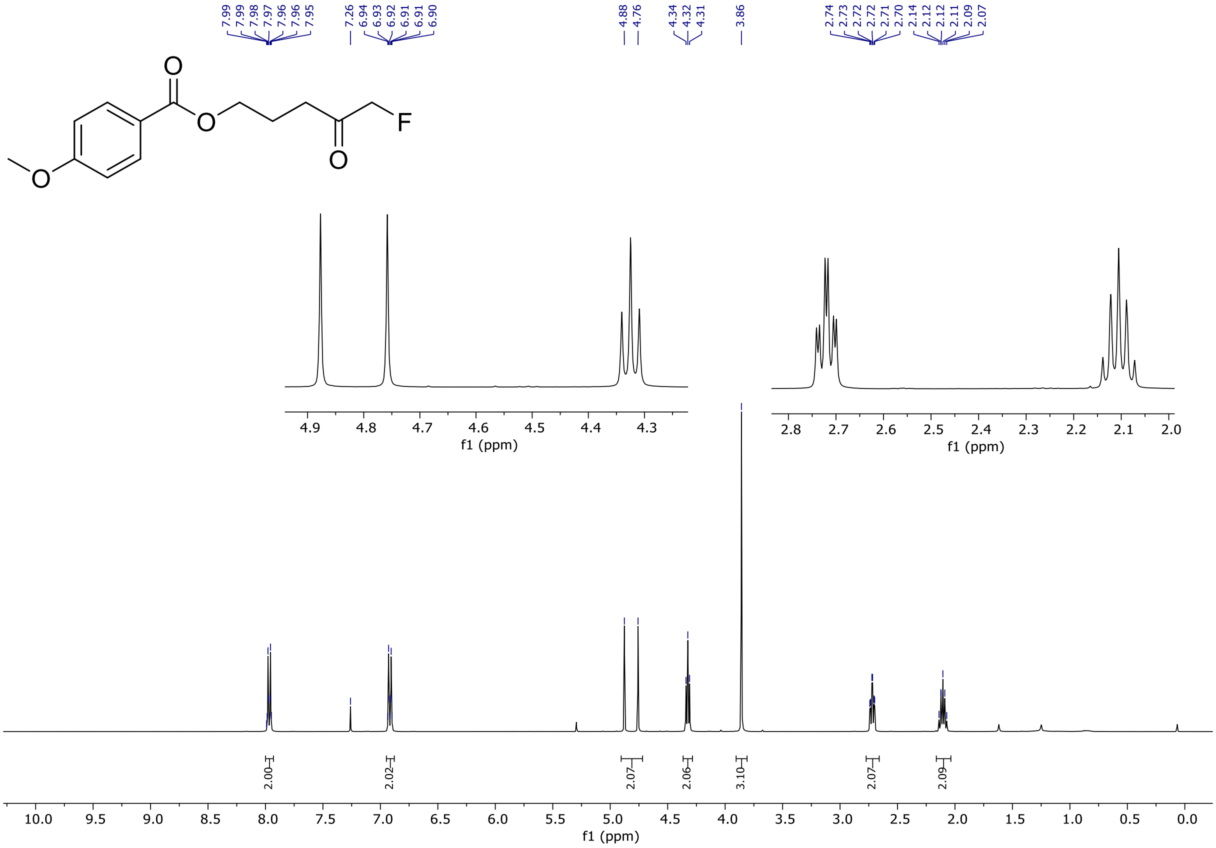


**$^{13}$C NMR** (101 MHz, $CDCl_3$, 299 K)

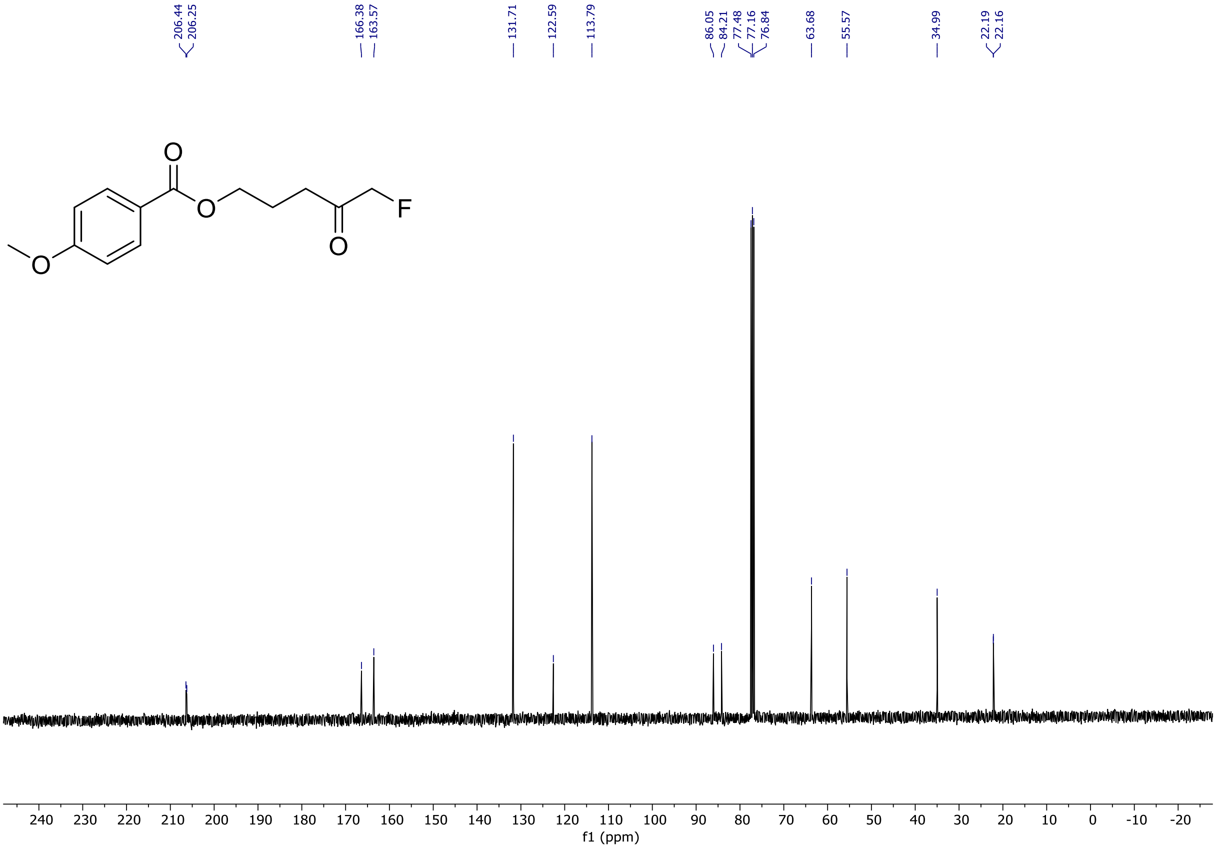

**$^{19}$F NMR** (376 MHz, $CDCl_3$, 299 K)

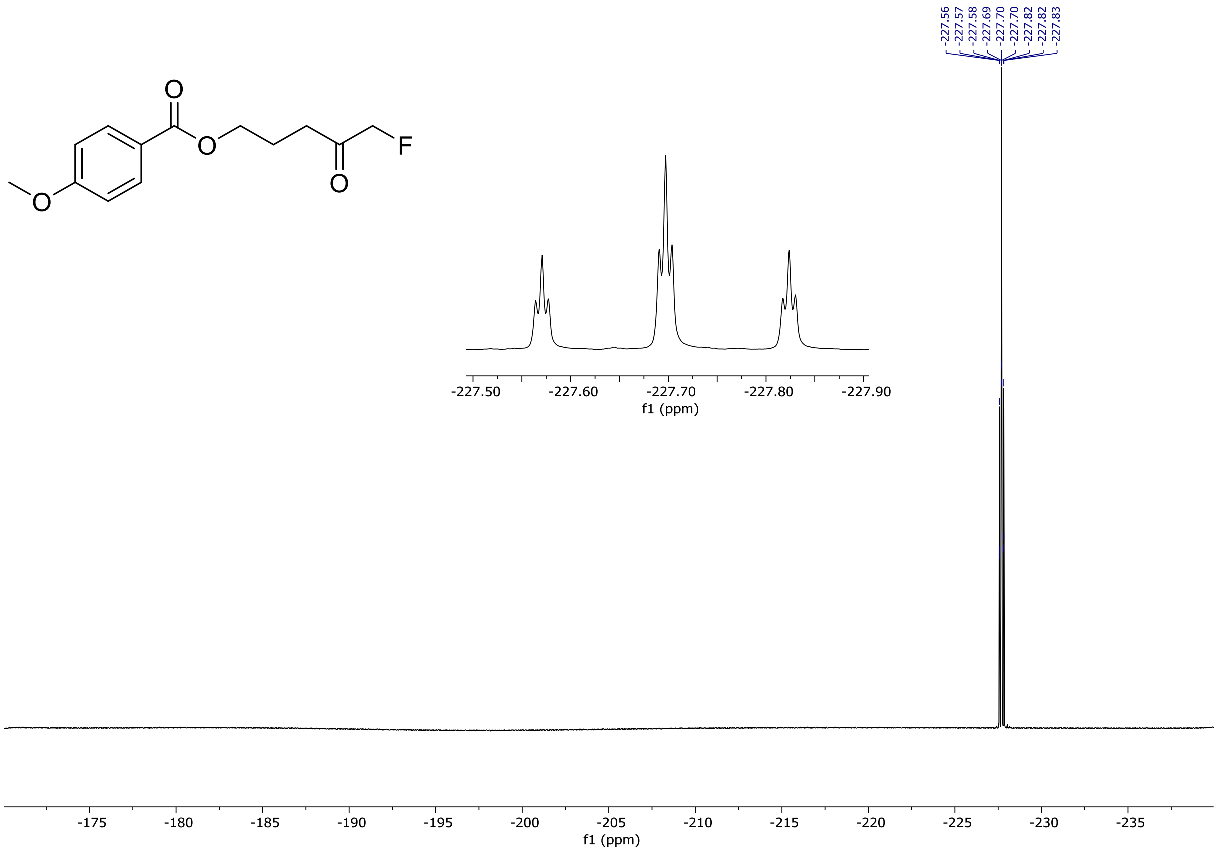

## 5-Fluoro-4-oxopentyl 4-nitrobenzoate (5)

**$^1$H NMR** (599 MHz, $CDCl_3$, 299 K)

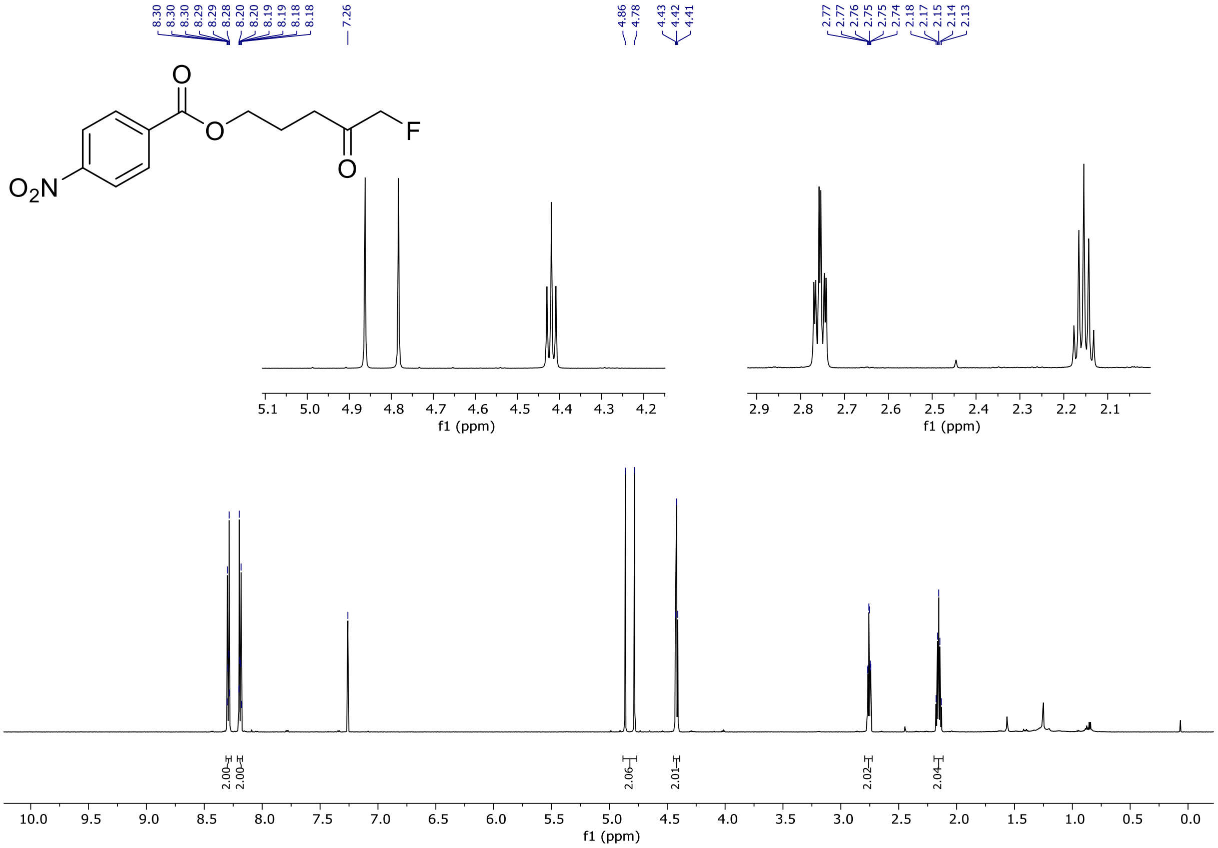


**$^{13}$C NMR** (151 MHz, $CDCl_3$, 299 K)

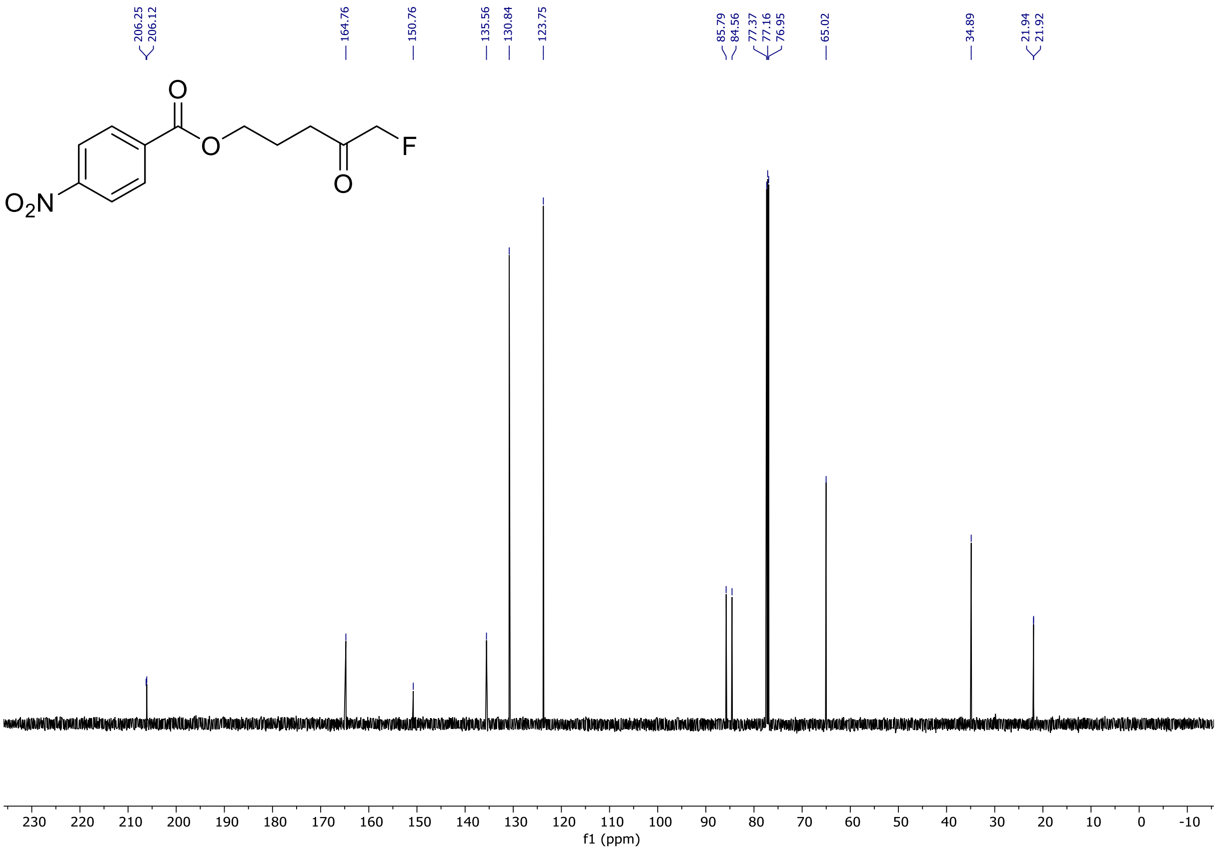

**$^{19}$F NMR** (564 MHz, $CDCl_3$, 299 K)

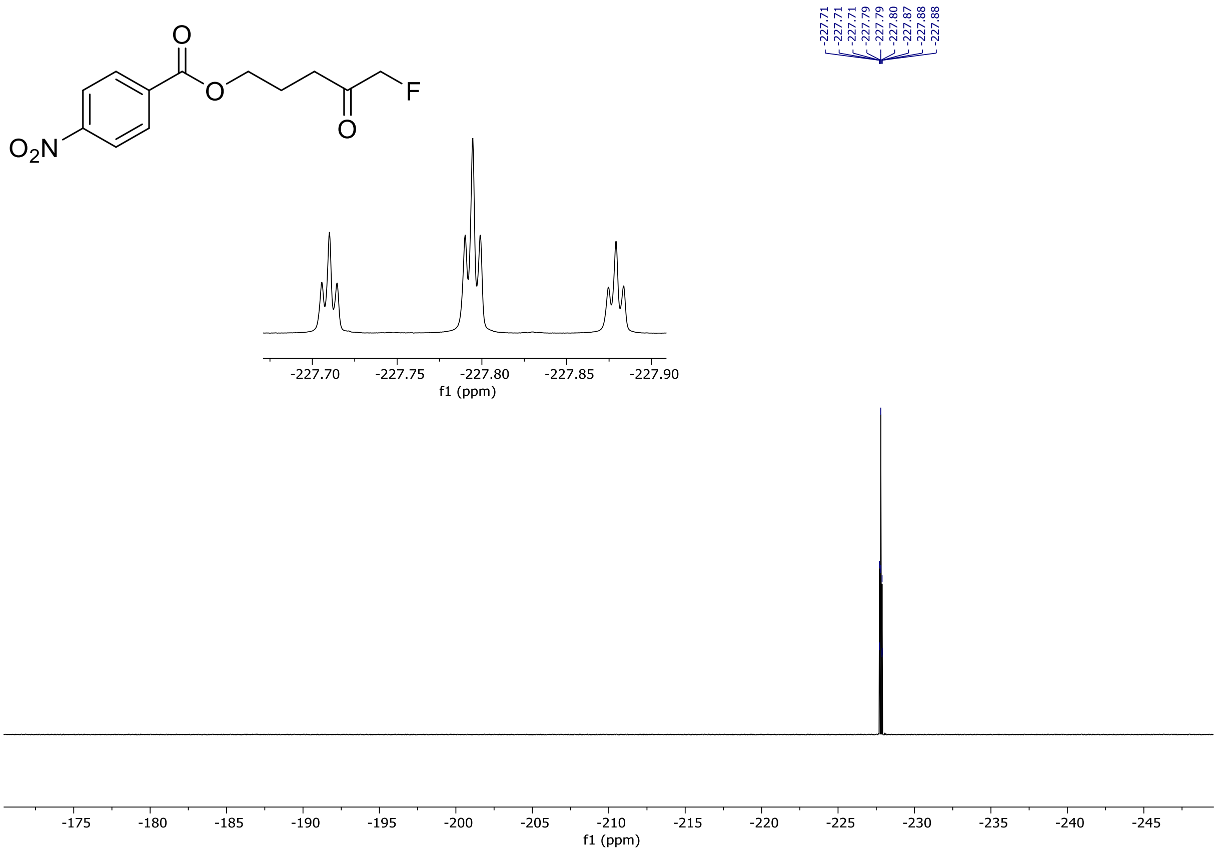

## 5-Fluoro-4-oxohexyl benzoate (6)

**¹H NMR** (500 MHz, CDCl₃, 299 K)

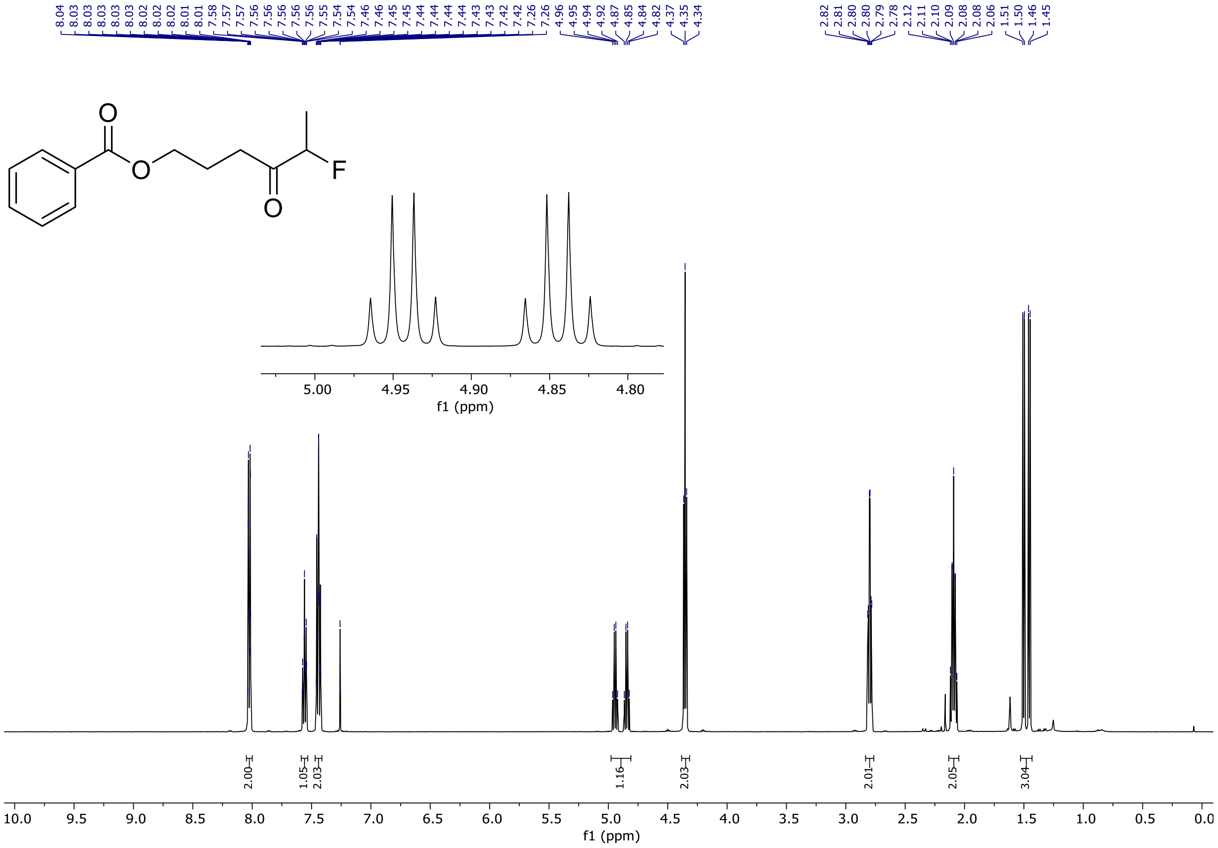

**¹³C NMR** (126 MHz, CDCl₃, 299 K)

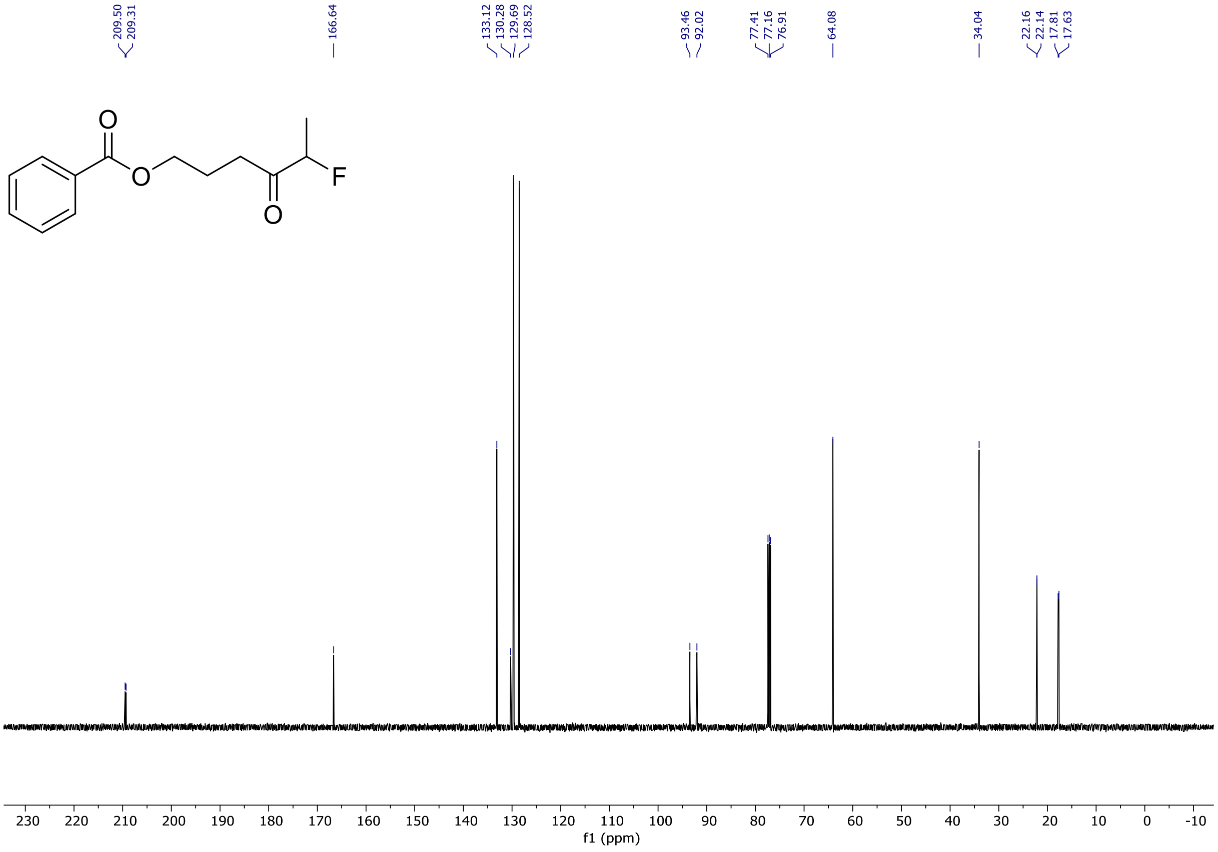

**$^{19}$F NMR** (470 MHz, $CDCl_3$, 299 K)

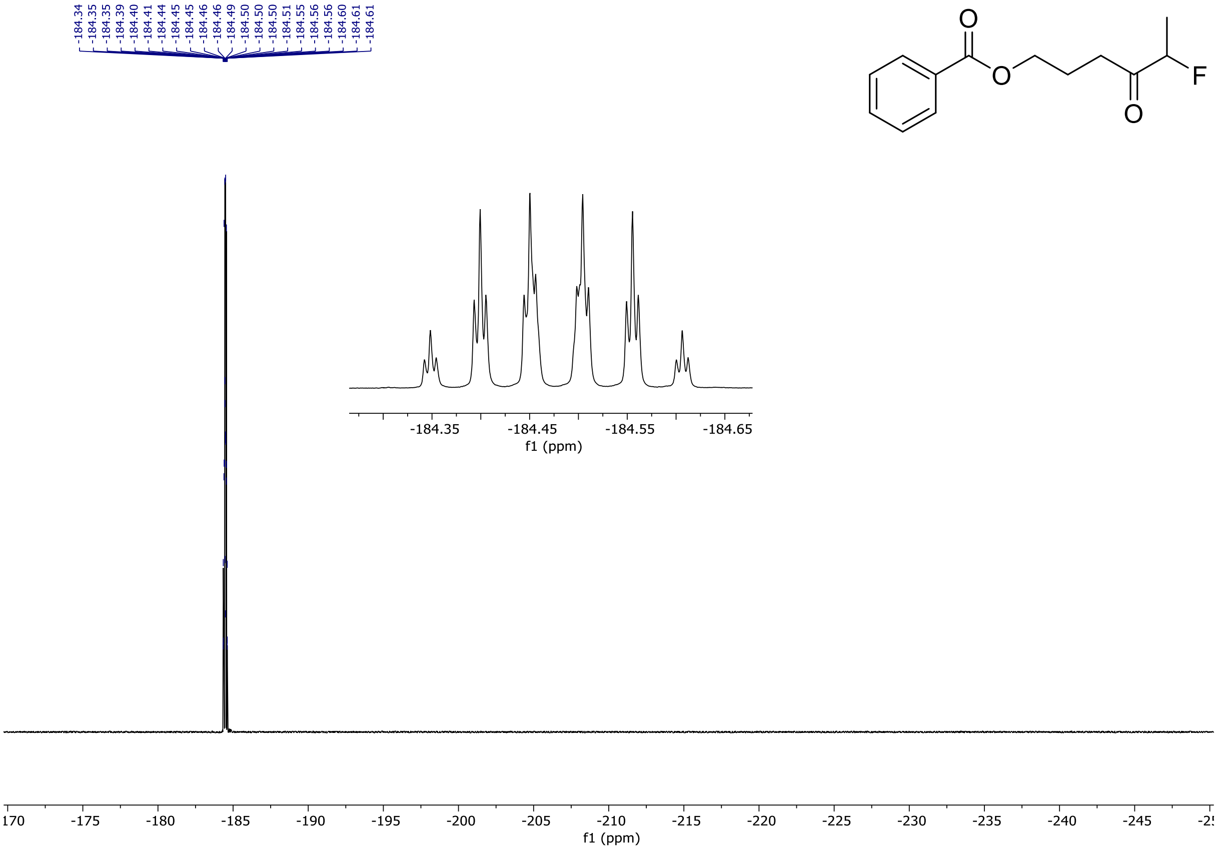

## 2-(5-Fluoro-4-oxopentyl)isoindoline-1,3-dione (7)

**$^1$H NMR** (500 MHz, $CDCl_3$, 299 K)

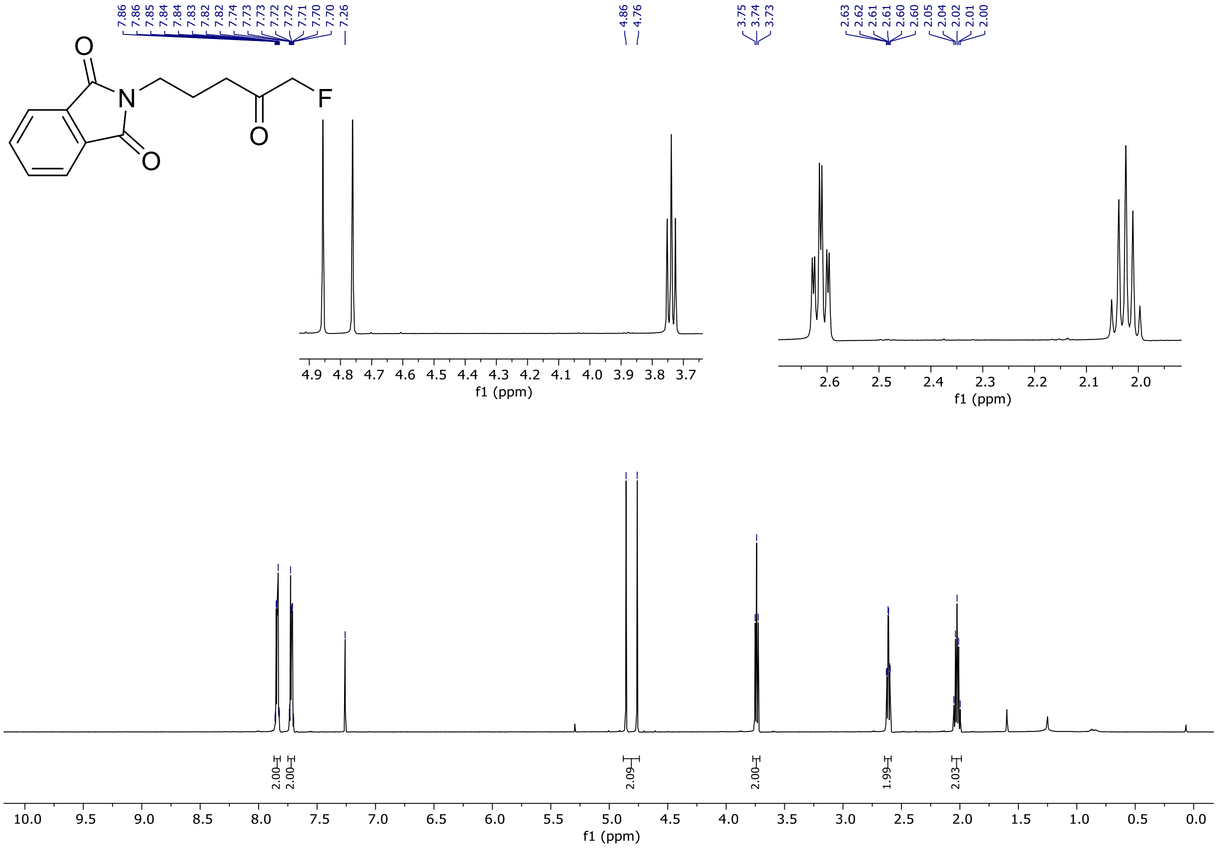

**$^{13}$C NMR** (126 MHz, $CDCl_3$, 299 K)

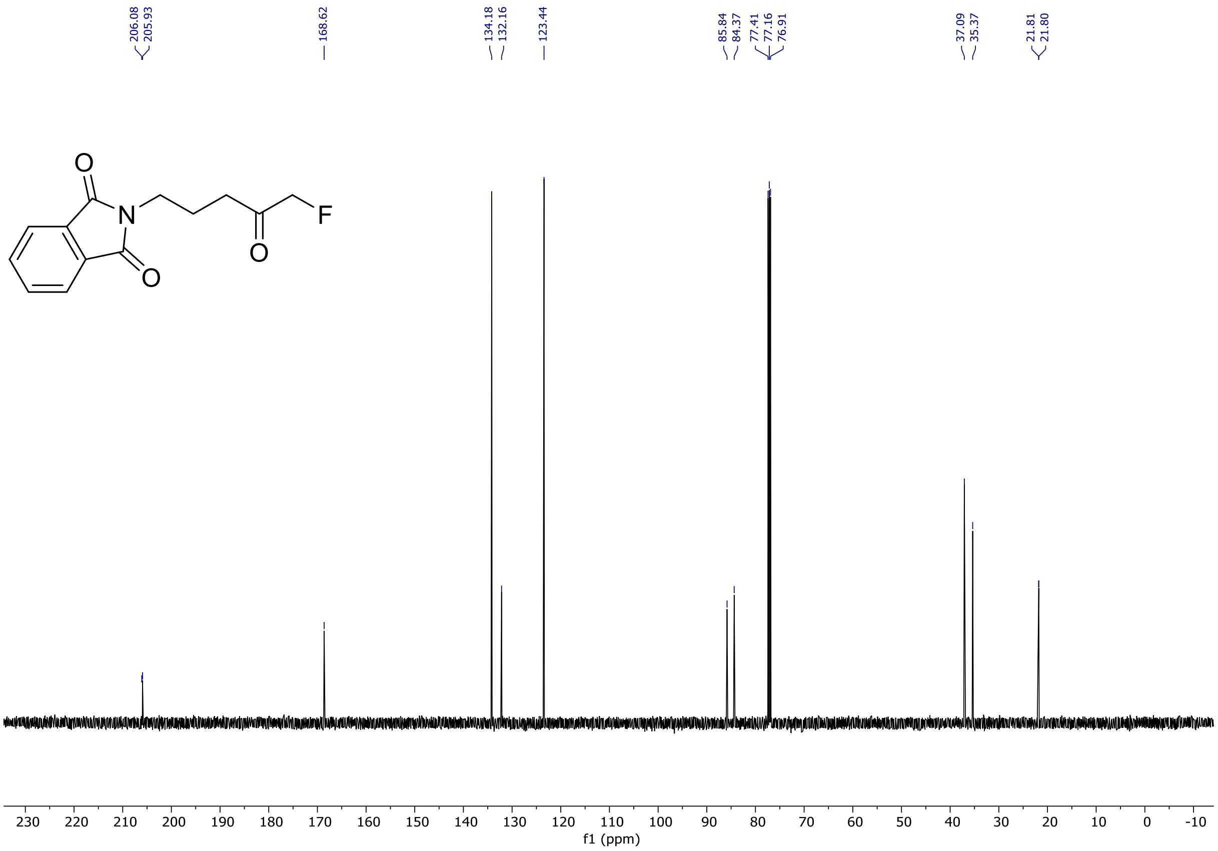

**$^{19}$F NMR** (470 MHz, $CDCl_3$, 299 K)

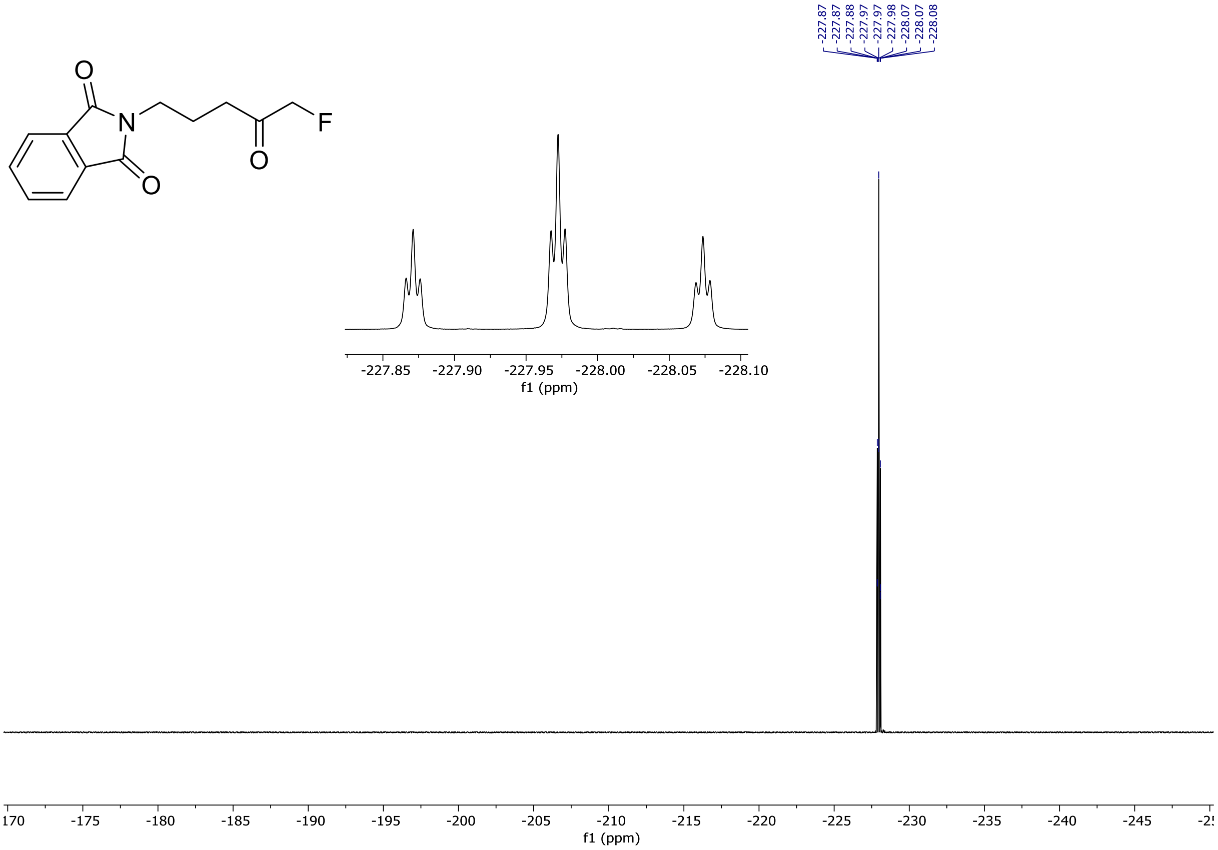

# X. References


1. Zhou, Y.; Wang, L.; Li, S.; Ma, S.; Walsh, P. J.; Bian, Q.; Li, F.; Zhong, J. *Synlett,* **2020**, *31*, 60-64.
2. Pareira, G. R.; Brandao, G. C.; Aramtes, L. M.; De Oliveira, H. A.; De Paula, R. C.; Do Nascimento, M. F. A.; Dos Santos, F. M.; Da Rocha, R. K.; Lopes, J. C. D.; De Oliveira, A. B. *Eur. J. Med. Chem.* **2014**, *73*, 295-309.
3. Xu, T.; Cheung, C. W.; Hu, X. *Angew. Chem. Int. Ed.* **2014**, *53*, 4910-4914.
4. Wu, W.; Dai, W.; Ji, X.; Cao, S. *Org. Lett.* **2016**, *18*, 2918-2921.
5. Hötling, S.; Haberlag, B.; Tamm, M.; Collatz, J.; Mack, P.; Steidle, J. L. M.; Vences, M.; Schulz, S. *Chem. Eur. J.* **2014**, *20*, 3183-3191.
6. Zheng, W. – F.; Xu, Q. – J.; Kang, Q. *Organometallics*, **2017**, *36*, 2323-2330.
7. Chen, Z.; Wen, Y.; Fu, Y.;Chen, H.;Ye, M.; Luo, G. *Synlett*, **2017**, *28*, 981-985.
8. Atobe, S. A.; Masuno, H.; Sonoda, M.; Suzuki, Y.; Shinohara, H.; Shibata, S.; Ogawa, A. *Tetrahedron Lett.* **2012**, *53*, 1764-1767.
9. Slack, E. D.; Gabriel, C. M.; Lipshutz, B. H. *Angew. Chem. Int. Ed.* **2014**, *53*, 14051-14054.
10. Gathirwa, J. W.; Maki, T. *Tetrahedron*, **2012**, *68*, 370-375.
11. Vechorkin, O.; Barmaz, D.; Proust, V.; Hu, X. *J. Am. Chem. Soc.* **2009**, *131*, 12078-12079.
12. Yu, T. – B.; Bai, J. Z.; Guan, Z. *Angew. Chem. Int. Ed.* **2009**, *48*, 1097-1101.
13. Quinones, N.; Seoane, A.; Garcia-Fandino, R.; Mascarenas, J. L.; Gulias, M. *Chem. Sci.* **2013**, *4*, 2874-2879.
14. Mateu, N.; Kidd, S. L.; Kalash, L.; Sore, H. F.; Madin, A.; Bender, A.; Spring, D. R.; *Chem. Eur. J.* **2018**, *24*, 13681-13687.

15. Sarie, J. C.; Thiehoff, C.; Mudd, R. J.; Daniliuc, C. D.; Kehr, G.; Gilmour, R. *J. Org. Chem.* **2017**, *82*, 11792-11798.
16. Ye, C.; Twamley, B.; Shreeve, J. M. *Org. Lett.* **2005**, *7*, 3961-3964.
17. Coplen, T. B.; Böhlke, J. K.; De Bièvre, P.; Ding, T.; Holden, N. E.; Hopple, J. A.; Krouse, H. R.; Lamberty, A.; Peiser, H. S.; Révész, K.; Rieder, S. E.; Rosman, K. J. R.; Roth, E.; Taylor, P. D. P.; Vocke, R. D.; Xiao, Y. K. *Pure Appl. Chem.* **2002**, *74*, 1987-2017.
18. Diakur, J.; Najashima, T. T.; Vedera, J. C. *Can. J. Chem.* **1980**, *58*, 1311-1315.
